# Comprehensive Genetic Dissection of the Hemocyte Immune Response in the Malaria Mosquito *Anopheles gambiae*

**DOI:** 10.1371/journal.ppat.1003145

**Published:** 2013-01-31

**Authors:** Fabrizio Lombardo, Yasmeen Ghani, Fotis C. Kafatos, George K. Christophides

**Affiliations:** Division of Cell and Molecular Biology, Department of Life Sciences, Imperial College London, London, United Kingdom; University of Minnesota, United States of America

## Abstract

Reverse genetics in the mosquito *Anopheles gambiae* by RNAi mediated gene silencing has led in recent years to an advanced understanding of the mosquito immune response against infections with bacteria and malaria parasites. We developed RNAi screens in *An. gambiae* hemocyte-like cells using a library of double-stranded RNAs targeting 109 genes expressed highly or specifically in mosquito hemocytes to identify novel regulators of the hemocyte immune response. Assays included phagocytosis of bacterial bioparticles, expression of the antimicrobial peptide CEC1, and basal and induced expression of the mosquito complement factor LRIM1. A cell viability screen was also carried out to assess dsRNA cytotoxicity and to identify genes involved in cell growth and survival. Our results identify 22 novel immune regulators, including proteins putatively involved in phagosome assembly and maturation (Ca^2+^ channel, v-ATPase and cyclin-dependent protein kinase), pattern recognition (fibrinogen-domain lectins and Nimrod), immune modulation (peptidase and serine protease homolog), immune signaling (Eiger and LPS-induced factor), cell adhesion and communication (Laminin B1 and Ninjurin) and immune homeostasis (Lipophorin receptor). The development of robust functional cell-based assays paves the way for genome-wide functional screens to study the mosquito immune response to infections with human pathogens.

## Author Summary

The mosquito immune system relies on innate humoral and cellular reactions to fight infections, including those by malaria parasites that must pass through mosquitoes before they can infect humans. Therefore, a detailed molecular understanding of these reactions could assist the design of new ways to control the spread of malaria and other mosquito-borne diseases. Here we use a technique to silence in mosquito cultured cells genes that are highly and/or specifically expressed in mosquito hemocytes, the equivalent of human white blood cells, as a means to investigate their function in reactions of the mosquito immune system. Our study identifies several novel regulators of immune reactions including phagocytosis, the engulfment and subsequent destruction of bacteria and other pathogens by hemocytes, the production of antimicrobial peptides, which directly kill or inhibit the proliferation of microbes, and the basal and induced production of an important complement regulator. Complement is a robust reaction of mosquitoes against malaria parasites and bacteria through phagocytosis, lysis or melanization (the enclosure of pathogens in a melanin capsule). We also reveal intriguing molecular connections between these reactions such as phagocytosis and regulation of complement. Our study provides novel insights into mosquito immune system and its reactions against infections.

## Introduction

*Anopheles gambiae* is a major vector of *Plasmodium falciparum* malaria in sub-Saharan Africa and a secondary vector of other parasitic and viral diseases [1]. Differences in vector susceptibility to malaria parasites are partly attributed to the ability of the mosquito immune system to fight infections. The developmental migration of *Plasmodium* within the mosquito hemolymph, the main carrier of the immune system, presents opportunities for the vector humoral and cellular immune reactions to attack the parasites [2]. Key functions of mosquito hemolymph components include killing of *Plasmodium* ookinetes as soon as they emerge from the midgut epithelium [3] and sporozoites before they invade the salivary glands [4]. Numerous mosquito agonist and antagonist effectors of *Plasmodium* and bacteria have been identified, principally by RNAi-mediated reverse genetic tests using dsRNA (double-stranded RNA) injections into adult mosquitoes [5]. These factors operate in complex molecular networks that involve pathogen recognition by secreted or membrane bound receptors, activation of immune signaling pathways, and synthesis or activation of effectors that contribute to lysis, melanization or phagocytosis of the invading pathogens [2], [6], [7]. Importantly, many of these factors are produced by hemocytes and function in the hemolymph [8], [9], [10]. Two hemocyte expression datasets have been reported recently, providing a comprehensive list of hemocyte-expressed genes [8], [11].

*An. gambiae* cell lines have been used extensively to study mosquito immune responses [12], [13], [14], [15]. Indeed, these cells are capable of accomplishing complex immune tasks that include phagocytosis of bacteria and beads [16], as well as expression of immune factors upon microbial challenge. It has been shown that IMD pathway activation in cell lines leads to robust expression of the antimicrobial peptide (AMP) gene *Cecropin 1* (*CEC1*) [15] and other immune factors [13]. This pathway is activated when the transmembrane receptor PGRPLC binds peptidoglycan (PGN) and induces nuclear translocation of the REL2 transcription factor [15], [17], [18].

The development of high-throughput RNAi screens in cultured cells has been a major breakthrough in functional genomics of model organisms, in both basic and applied research [19], [20], [21], [22]. Here we report the development and implementation of RNAi screens in *An. gambiae* cells to provide insights into the functional immune repertoire of mosquito circulating hemocytes. We have generated a dsRNA library targeting 109 genes specifically or predominantly expressed in circulating hemocytes and then optimized cell-based RNAi screens to investigate the role of these genes in phagocytosis of bacteria and transcriptional activation of immune-related genes. Our results identify novel regulators of the hemocyte immune responses and interactions with pathogens, including regulators of a complement-like pathway component that plays a key role in reactions to malaria parasites. This is a key milestone towards development of genome-wide RNAi screens in *An. gambiae* cells.

## Results/Discussion

### A hemocyte-specific dsRNA library

We generated a set of 111 dsRNAs to target 109 genes that exhibit enriched expression in hemocytes, differential regulation by immune challenges, and presence of immune-related InterPro domains and/or signal peptide or transmembrane domains (Dataset S1). To populate and annotate this dsRNA library we used: two published datasets of genome-wide transcriptional repertoires of *An. gambiae* circulating hemocytes from naive or *Plasmodium* infected adult females [8], [11], the published expression profile of *An. gambiae* hemocyte-like cell lines in response to microbial challenges [13], the VectorBase *An. gambiae* genome annotation [23] and information about the silencing phenotypes of *Drosophila* orthologs found in the GenomeRNAi database [24], [25]. Analysis of AMP expression and dsRNA-mediated RNAi efficiency (Text S1) led us to choose the Sua5.1* cell line [12], [16], [26] as our model experimental system.

### Cell growth and viability

We carried out viability screens to assess the levels of dsRNA toxicity as determined by the effect of gene silencing on fundamental housekeeping processes such as cell growth, proliferation and survival, which could hamper true identification of immune regulators. *An. gambiae* homologs of three genes previously shown to cause lethal or growth-defective RNAi phenotypes in *Drosophila* cells [22], [27] were used as controls: inhibitor of apoptosis 1 (IAP1; AGAP007294), a ubiquitin-like/ribosomal fusion protein (AGAP008001; 2 different dsRNA fragments) and the Rho1 small GTPase (AGAP005160) (Table S2 and Text S1).

Two protocols were implemented to assess cell growth and viability: (a) image acquisition and quantification by automated fluorescence microscopy, which is time-consuming and technically challenging but allows for more accurate and informative analysis; and (b) microplate reader fluorescence quantification, which is quicker and can accommodate large datasets but is less user-responsive.

**Figure 1:**
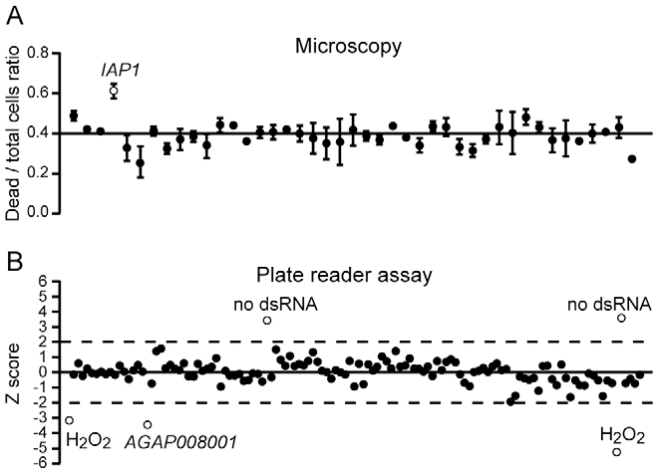
Cell growth and viability screen. (A) A fraction of the dsRNA hemocyte-specific library was screened twice by automated fluorescence microscopy. Fluorescent cells were automatically counted using protocols developed in Volocity Improvision and ImageJ software, as described in Materials and Methods. The ratios of dead cells (Sytox Green positive) over the total number of cells (Hoechst positive), and the standard deviation of replicates, are shown. (B) The entire dsRNA collection was screened using the Cell Titer Blue/Plate Reader method. The plot shows the z-score analysis of one representative set of experiments. A z-score threshold of at least 2 was chosen, and positive hits are shown as open circles. No dsRNA-treated samples and samples treated with 100 mM H_2_O_2_ are also indicated. Three biological replicates were performed.

A subset of 37 dsRNAs was screened by automated fluorescence microscopy. Staining cells with Sytox green/Hoechst was used to assess the effect of dsRNAs on cell viability. Protocols in Volocity Improvision and ImageJ softwares were developed to capture images and quantify fluorescent cells, to determine the ratio of dead cells (Sytox green positive) over the entire cell population (Hoechst positive) (Figure 1A and Materials and Methods). ANOVA statistics followed by Bonferroni's post-test correction revealed that ds*IAP1* leads to a significant increase of cell mortality. Silencing *Rho1* significantly reduced the number of cells but did not increase cell mortality (Figure S1, Figure S2 and Text S1). The cells and their nuclei were much larger in size indicating a defect in cytokinesis and cell cycle progression. A similar phenotype was observed in Drosophila S2 cells after silencing the orthologous *Rho1* gene [22], [27].

Next, we screened the entire collection of 111 dsRNAs using plate reader quantification in conjunction with a CellTiter-Blue Cell Viability assay. Data from three replicates were analyzed and z-score analysis was performed posing thresholds of +/−2 for at least two replicates out of three. Reproducibility among replicates was evaluated by correlation tests as shown in the plots in Figure S3. Silencing the ubiquitin-like/ribosomal fusion protein gene *AGAP008001* led to significant decrease in cell viability (Figure 1B and Figure S3). No other dsRNA treatment resulted in statistically significant deviation from the average cell count. A general observation was that dsRNA treatment resulted in significant cell mortality that was independent of the targeted gene.

We validated the viability data *in vivo* by injecting dsRNAs of control *LacZ*, *IAP1* and *AGAP008001* into newly emerged adult female mosquitoes and then monitoring the survival of KD mosquitoes daily (Figure S1). Both gene KDs led to statistically significant increase of mortality rates compared to control. In addition to the support that this analysis provided to our experimental approach, the good correspondence between the *ex vivo* cell-based viability screen and the *in vivo* phenotypic analysis highlights opportunities for future use of such viability screens in identifying targets of novel mosquito insecticides.

### Bacterial phagocytosis screens

Phagocytosis is a highly effective and immediate response against microbial invaders [28]. Mosquito hemocytes can bind and phagocytose bacterial bioparticles and Sephadex beads, as well as malaria sporozoites [4], [29], [30], [31]. We established a fast and reliable cell-based assay in *An. gambiae* cells using *Escherichia coli* bioparticles conjugated with pHrodo succinimidyl ester, a pH-sensitive fluorescent dye, to investigate the potential role of genes in the hemocyte-enriched library in bacterial phagocytosis. The phagocytic activity of cells was determined as the increase of bioparticle fluorescence caused by the drop of pH in the acidified phagosomes [32].

**Figure 2:**
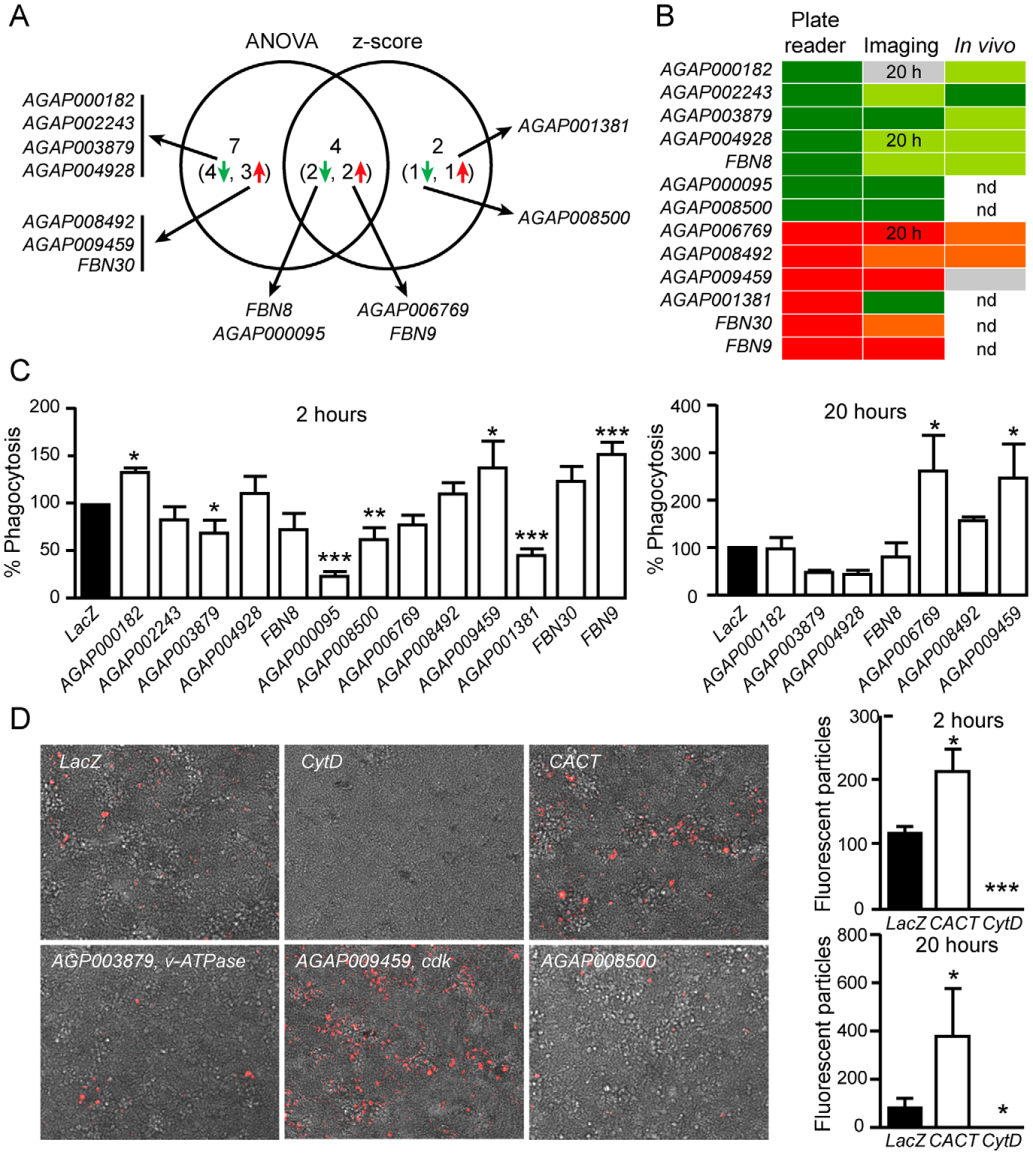
Bacterial phagocytosis screen. (A) Venn diagram showing the results of the z-score and ANOVA analyses of data obtained with the microplate reader phagocytosis assay. (B) Heat map depicting the performance in the microscopy imaging and *in vivo* phagocytosis assays of the 13 dsRNAs identified as positive hits with the microplate reader assay. Dark green, significant decrease of phagocytosis; green, decrease of phagocytosis; dark red, significant increase of phagocytosis; red, increase of phagocytosis; grey, similar to LacZ control; nd, not determined. (C) Microscopy imaging analysis: phagocytosis rates of cells at 2 and 20 h following bioparticles challenge when genes identified as modulators of phagocytosis by microplate reader assay are silenced. Data are shown as percent phagocytosis compared to ds*LacZ*-treated controls. (D) Examples of fluorescence microscopy images of Sua5.1* cells treated with dsRNAs or Cytochalasin D. Images were captured 20 h after challenge with pHRodo bioparticles. The graphs indicate the capacity of cells to uptake bioparticles following ds*LacZ*, ds*Cactus* and Cytochalasin D treatments at 2 and 20 h after challenge, as quantified by image analysis. Mean values of counted particles and standard errors are reported. Results from two experiments are shown. Asterisks indicate statistically significant differences between each KD and the dsLacZ-treated controls (*: P<0,05; **: 0,005<P<0,05; ***: 0,0005<P<0,005).

Bacterial bioparticles were added to the cells three days after incubation with dsRNA, and the capacity of cells to uptake bioparticles was assessed by fluorescence measurements using a microplate reader. Four time-points were assayed to take into account the kinetics of bioparticle uptake (1, 3, 6 and 24 h post-challenge). The measurements obtained were subtracted from basal level measurements (0 h, immediately after challenge). The entire dsRNA library was screened three times and z-score values for each of the dsRNA in each of the replicate screens were calculated. We considered positive dsRNA hits those with z-score values above 2 or below −2 in at least two out of the three replicates for each time point (Figure S4 and Supplemental Table S3). Because our library is strongly biased towards genes that are likely to play a role in immune reactions, the z-score method which compares the effect of each dsRNA with the average effect of all dsRNAs is a very strict condition. Therefore, we also analysed the data using ANOVA followed by Tukey's multiple comparison test, thus comparing each dsRNA with the reference ds*LacZ* control. The results from both methods revealed a total of 13 positive dsRNA hits, 6 from the z-score and 11 from ANOVA (Table S3 and Figure 2A–B). Four dsRNAs showed significant effects on bioparticle uptake with both methods: 2 of them decreased phagocytosis (*FBN8* and *AGAP000095*) and 2 of them increased phagocytosis (*AGAP006769* and *FBN9*). *Cactus* dsRNA that was used as a positive control also led to a significant increase in phagocytosis of *E. coli* as soon as 1 h after challenge, in consistence with previous observations [29].

We examined the 13 positive hits from the microplate reader analysis using automated fluorescence microscopy and a protocol for quantification of phagocytosed bioparticles developed in the ImageJ software. Bioparticle uptake was monitored 2 h and 20 h after challenge and compared to the ds*LacZ* control using ANOVA (Figure 2C–D). An overall consistency was observed between the microplate reader and the microscopy analyses. Of the 13 dsRNAs, 6 showed the expected phenotype with statistical significance, 5 showed the expected phenotype but were not statistically significant, and 2 showed no difference with the ds*LacZ* control and/or a phenotype opposite to the expected, respectively (Figure 2B). As mentioned earlier, imaging analysis can provide additional, more detailed, information when compared to the microplate reader method, but it is technically more challenging and time consuming. The few discrepancies between these two approaches may be due to both technical and biological reasons, for example image analysis cannot quantify the amount of bioparticles in a single cell, while the microplate reader quantifies the intensity of fluorescence. Moreover, as shown in Figure 2D, the distribution of fluorescent bioparticles is not uniform in the cell layer, and this introduces another variable when microscope images are captured and analyzed.

**Figure 3:**
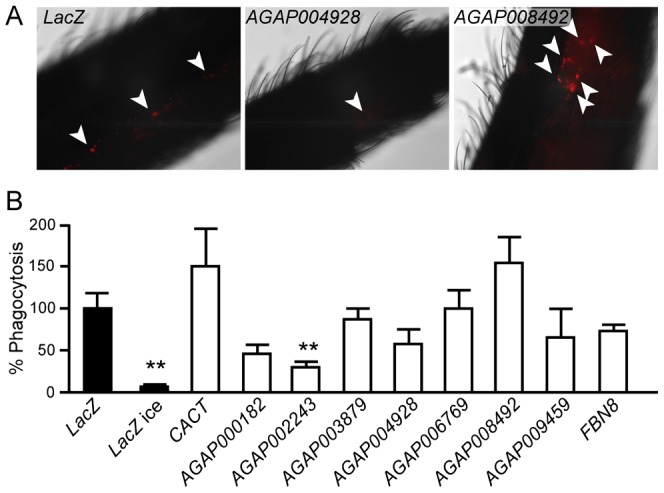
*In vivo* phagocytosis assay. (A) Pictures of abdominal segments of adult female mosquitos injected with dsLacZ, dsAGAP004928 and dsAGAP008492 and then challenged with pHRodo *E. coli* bioparticles. Fluorescence and brightfield images were captured and hemocytes containing fluorescent bioparticles quantified. The experiment was repeated twice with 8–10 mosquitoes per treatment. Quantification of fluorescent bioparticles and statistical analysis were performed as described in Materials and Methods. (B) The graph shows the percentage of phagocytosis of the different KDs placing dsLacZ as reference. Mean values and standard errors are reported. Asterisks indicate statistical significance (**: 0,005<P<0,05) according to t-tests applied to each gene KD compared to *LacZ*KD.

Next, we investigated the silencing effect of 8 out of the 13 dsRNAs identified by the microplate reader method on bacterial phagocytosis *in vivo*. For this, we employed a protocol that was used in an earlier study, in which bacteria injected into the mosquito hemolymph spread rapidly into the cavity and are phagocytosed by hemocytes often found in clusters associated with the tracheal system [29]. We injected 2-day old mosquitoes with dsRNAs and 4 days later re-injected them with *E. coli* pHRodo-conjugated bioparticles. Mosquitoes were dissected 1 h after bioparticle injection, mounted onto glass slides and immediately observed by fluorescence microscopy. Pictures of different parts of the mosquito abdomens were captured and analyzed using a protocol developed in ImageJ to quantify the numbers of fluorescent particles (Figure 3). The results revealed strong *in vivo* phenotypes similar to those of the cell-based analysis for 4 out of the 8 dsRNAs: AGAP000182, AGAP008492, AGAP004928 and AGAP002243, the last one corroborated by a significant statistical evaluation. Three dsRNAs (AGAP003879, FBN8 and AGAP006769) also showed similar albeit weaker phenotypes compared to the cell-based analysis and only one dsRNA (AGAP009459) did not confirm the expected phenotype.

### Novel regulators of phagocytosis

Based on to the microplate reader analysis, the silencing of 7 genes led to a significant decrease of the cellular capacity to phagocytose *E. coli* bioparticles. Some of these genes were also confirmed with the microscopy and the *in vivo* analysis, as presented above. These genes encode: a protein of unknown function with a putative signal peptide and a peptidase domain (*AGAP000182*); a protein with a homodimerization BTB/POZ domain, ankyrin repeats and a zinc finger domain (*AGAP002243*); a putative transmembrane v-ATPase (*AGAP003879*); a membrane-bound protein with a zinc finger and a LITAF (LPS-induced tumor necrosis factor alpha factor) domain putatively involved in immune signaling (*AGAP004928*) [33], [34]; the fibrinogen-domain FBN8 (also known as FREP57), previously shown to play a role in anti-*Plasmodium* defense (*AGAP011223*) [35]; a putative Calcium channel protein (*AGAP000095)*; and a three-transmembrane protein of unpredicted function (*AGAP008500*).

Two of these proteins are likely to play a role in phagosome formation and maturation/acidification. The putative Ca^2+^ channel protein (AGAP000095) may be involved in the cellular Ca^2+^ balance that is required for solubilization of the actin meshwork surrounding nascent phagosomes, fusion of phagosomes with granules containing lytic enzymes, or assembly and activation of the superoxide-generating NADPH oxidase complex [36]. The v-ATPase (*AGAP003879*) is known to play a role in phagosome acidification in other model organisms [37]. Orthologs of *AGAP003879* and *AGAP004928* in *D. melanogaster* show similar phenotypes in RNAi screens that investigate host-pathogen interactions (Dataset S1), as both KDs cause a decrease in intracellular *Listeria monocytogenes* infection [38].

In contrast, silencing 6 out of the 109 genes leads to a significant increase of *E. coli* phagocytosis. Proteins encoded by these genes represent potential novel negative regulators of bacterial recognition and phagosome assembly. These include: a secreted protein of unknown function that is strongly expressed in mosquito hemocytes [8] and cultured cells following LPS or PGN challenge (*AGAP006769*) [13]; a putative tyrosine and serine/threonine kinase (*AGAP009459*) with homologs described in other mosquitoes (*Aedes aegypti*, *AAEL008621*, cell division protein kinase 1, cdk1; and *Culex quinquefasciatus*, *CPIJ001155*, cdk2) and *D. melanogaster* (CG5363, cdc2 cell division control protein); *AGAP008492* that does not exhibit similarity to any other genes and is regulated during immune challenges [13]; the ortholog of *Drosophila* laminin B1 chain (*AGAP001381*); and the fibrinogen-domain lectins FBN30/FREP8 and FBN9/FREP13 (*AGAP006914* and *AGAP011197*, respectively) [35].

The negative effect of *AGAP009459* silencing in bacterial phagocytosis is possibly related to defects in cytoskeleton regulation. Its *Drosophila* ortholog, cdc2, is similarly involved in defense-related processes as highlighted by increased *Listeria* intracellular infection, reduced *Chlamydia* infection and decreased *Drosophila* C virus and influenza virus replication following silencing [38], [39], [40]. Similarly, the fruit fly ortholog of Laminin B1 may also play a role in innate immune reactions since its silencing is shown to decrease viability after intestinal infections with *Serratia marcescens* [41]. FBN9 has been previously shown to be upregulated both by malaria parasite and *E. coli* infections [42]. The involvement of FBN9 in the defense against bacteria and maintenance of basal immune homeostasis is supported by evidence that the protein is found on the surface of non-challenged cells and strongly co-localizes with bacteria as well as malaria parasites following infection [35]. A specific role of FBN9 as a negative regulator of phagocytosis can be therefore hypothesized considering the fine interplay between different immune processes, where a pattern recognition receptor may specifically promote one in favor of another process.

### Transcriptional activation of the AMP *CEC1*

**Figure 4:**
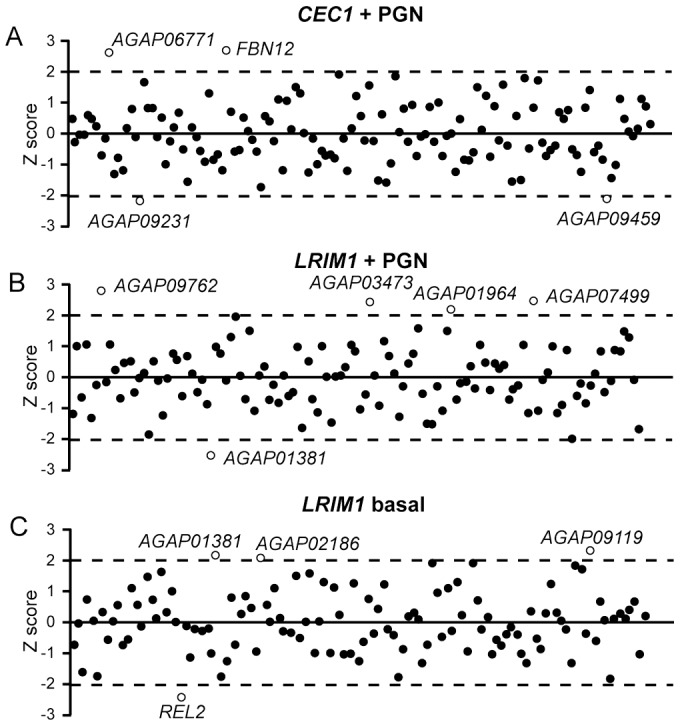
Luciferase assays. (A) Transcriptional activation of *CEC1* promoter upon PGN challenge. The graph reports the z-scores calculated from the ratios of averaged RLU measurements after PGN and PBS treatments. (B and C) Transcriptional regulation of LRIM1 promoter (B) upon PGN challenge (z-scores calculated from the ratios of averaged RLU measurements after PGN and PBS treatments) and (C) in basal conditions (z-scores calculated from the RLU measured after PBS treatments). Positive hits are shown as open circles.

It has been previously shown that the AMP *CEC1* is transcriptionally induced in cultured cells following immune challenge and that this induction depends partly on the IMD signaling pathway [13], triggered after PGN recognition by the PGN Recognition Protein LC (PGRP-LC) [18]. To identify novel hemocyte regulators of the *A. gambiae* IMD pathway and potentially other pathways regulating the expression of AMPs, we developed a luciferase reporter assay in Sua5.1* cells to screen the hemocyte dsRNA library. A 660 bp fragment of the *CEC1* promoter cloned upstream of the *luciferase* gene was used [15]. Initial experiments revealed a significant induction of *CEC1* promoter activity 7 h after PGN challenge. A dsRNA targeting the NF-κB transcription factor REL2, previously shown to regulate *CEC1* transcriptional activation [15], was included as a positive control. Changes in luciferace activity were analyzed by calculating the z-score values of ratios of average RLU (Relative Light Units) measurements of PGN vs. PBS treatments (Figure 4 and Table S4). One-way ANOVA followed by Dunnet's multiple-comparison post-test was also performed to compare ds*LacZ* control and KD values (Table S4).

Silencing *AGAP010531* (*FBN12* or *FREP2*) and *AGAP006771* led to increased *CEC1* expression following PGN challenge. *AGAP006771* encodes a putative transmembrane protein orthologous to the *Drosophila* Tumor Necrosis Factor-like, eiger. Interestingly, *Drosophila* eiger is also induced during microbial infections and required for both resistance and tolerance to infections, partly by controlling the expression of the AMP Diptericin [33], [43]. Eiger functions as a negative regulator of AMP expression following PGN challenge and IMD pathway activation by blocking the expression of the NF-κB factor, Relish (the ortholog of REL2), through the JNK pathway [44]. Our data is consistent with the fruit fly model and identify mosquito eiger as a negative regulator of *CEC1* expression. Like FBN9 that is identified as a negative regulator of phagocytosis, FBN12 belongs to a family of putative pattern recognition receptors known to be involved in immune responses and maintenance of homeostasis; however, in contrast to FBN9, FBN12 is downregulated during bacterial infections [17], [35]. This is consistent with a role of FBN12 as a negative regulator of *CEC1* expression.

Silencing *AGAP009231* and *AGAP009459* reduced PGN-induced *CEC1* transcriptional activation. *AGAP009231* encodes a transmembrane domain protein of the family of ninjurins, most likely of the A sub-family, a complex class of cell adhesion molecules that are proteolytically processed and shed by matrix metalloproteases (MMP). *MMP1* and *NinjA* genes are co-expressed and upregulated in *D. melanogaster* S2 cells after LPS challenge [45] and in adult flies after wounding [46]. Proteolytic cleavage of NinjA by MMPs releases an ectodomain involved in cell adhesion and cell-cell communication [47]. MMPs are known to play various roles in inflammation and innate immunity but the identity and function of their substrates and mechanisms are still to be elucidated [48]. *AGAP009231* has been previously shown to be highly expressed and localized on the membrane of *An. gambiae* circulating hemocytes [8]. Our data showing involvement of this protein in AMP expression suggest an important role in mosquito innate immunity, probably in signaling and cell communication.

As discussed earlier, *AGAP009459* encodes the ortholog of *Drosophila* cyclin-dependent protein kinase cdc2 that has been implicated in several processes from cell cycle regulation to cytoskeleton remodeling. Importantly, RNAi silencing of *cdc2* also leads to decreased STAT92E phosphorylation, suggesting a regulatory role of cdc2 in JAK/STAT signaling [49]. The involvement of JAK/STAT pathway in fruit fly hemocyte differentiation and proliferation is well documented, but its exact role in immune responses such as AMP expression remains unclear [50]. In mosquitoes, the JAK/STAT pathway is activated by immune challenges and is involved in responses against pathogens [51], [52], [53], and our data suggest involvement of the JAK/STAT pathway in *CEC1* activation. The function of *AGAP009459* in phagocytosis of *E. coli* bioparticles may be related to the involvement of cdc2 in cytoskeleton regulation, but could also imply a novel role of JAK/STAT in phagocytosis.

### Transcriptional regulation of the complement factor LRIM1

We investigated the expression of the *LRIM1* gene using an approach identical to that described above for *CEC1*. *LRIM1* is expressed in *An. gambiae* hemocytes [8] and secreted in the hemolymph in a disulfide-linked complex with the structurally related protein APL1C; there the complex binds and solubilizes TEP1_cut_, a cleaved, activated form of the complement C3-like factor TEP1 [54], [55]. This new complex plays a key role in mosquito responses against invading malaria parasites. A previous study showed that *LRIM1* is transcriptionally induced following PGN challenge [13]. We used a 1600 bp fragment of the LRIM1 promoter fused to luciferase (courtesy of M.J. Povelones). Our preliminary data showed high luciferase activity in Sua5.1* cells but no further upregulation following PGN challenge.

We investigated whether the lack of *LRIM1* promoter upregulation in Sua5.1* cells upon PGN challenge was due to inhibition by other hemocyte factors (Figure 4 and Table S4). Our screen identified four genes that inhibit transcriptional activation of the *LRIM1* promoter following PGN challenge, as silencing *AGAP009762*, *AGAP007499*, *AGAP003473* and *AGAP001964* led to increased luciferase expression compared to PBS control. *AGAP009762* is highly expressed in circulating hemocytes [8] and encodes a EGF-like domain protein with similarities to *Drosophila* phagocytosis receptors eater [56] and NimC1 [57] as well as to other members of the Nimrod superfamily [58]. Interestingly, a screen for novel regulators of JNK following IMD pathway activation upon challenge with PGN in *Drosophila* cells, revealed that the ortholog of AGAP009762 caused an increased P-JNK protein expression, which, in turn, may act to modulate the expression of Relish-controlled effectors [59].

*AGAP007499* encodes a chloride channel protein, orthologous to the human chloride channel 7. It is strongly expressed in circulating hemocytes [8] and upregulated in mosquito cultured cells after hydrogen peroxide treatment [13]. The *Drosophila* ortholog of *AGAP007499* is shown to play a role in the receptor tyrosine kinase (RTK)-Ras-extracellular signal-regulated kinase (MAPK/ERK) signaling pathway; its silencing leads to increased MAPK phosphorylation following EGF stimulation [60]. It has been previously shown that MAPK ERK signaling plays a role in the mosquito immune response against malaria parasites [61]. *AGAP003473* encodes a transmembrane protein with no significant similarity to known proteins. Finally, *AGAP001964* encodes a previously uncharacterized member of the clip-domain serine protease subfamily A (CLIPA) that lacks protease activity. Importantly, several CLIPAs show *Plasmodium* infection phenotypes [62], [63], mostly by regulating the hemocyte-mediated melanization reaction. Since *LRIM1* is involved in malaria parasite melanization and lysis, as well as in bacterial phagocytosis, we hypothesize that the identified proteins function as negative regulators of these reactions some of which (e.g. melanization) are potentially costly to the host; thus *LRIM1* is induced only when these proteins are downregulated or presumably depleted during these reactions.

Silencing *AGAP001381* had an opposite effect, reducing luciferase expression driven by the *LRIM1* promoter 7 h after challenge with PGN compared to mock PBS challenge. As mentioned previously, *AGAP001381* encodes the ortholog of the fruit fly Laminin B1 and its silencing also increases phagocytosis of *E. coli* bioparticles. These data conform to our hypothesis that a network of negative and positive regulators is involved in induction of *LRIM1* expression that follows infection. Intriguingly, silencing of Laminin B1 also resulted in a contrasting increase of the basal *LRIM1* promoter activity (Figure 4C), suggesting a dual role of laminin B1 in activating and suppressing *LRIM1* expression in the presence and absence of immune challenge, respectively.

Two additional genes were identified as negative regulators of *LRIM1* basal expression: silencing of *AGAP009119* and *AGAP002186* led to a significant increase of luciferase expression driven by the *LRIM1* promoter (Figure 4C). *AGAP009119* encodes a protein with tetratrico peptide structural repeats, involved in protein-protein interactions, and a heat shock chaperonin-binding motif, which is orthologous to the *D. melanogaster* Hsp70-interacting protein 2 (HIP-replacement). *AGAP002186* encodes a receptor of lipophorin (Lp), that is the insect equivalent of low-density lipoproteins and co-ortholog of the *Drosophila* LpR1 and LpR2 [64]. In *An. gambiae*, Apolipoprotein I and II, the main components of Lp, have been shown to act antagonistically to TEP1-dependent malaria parasite killing [65], [66], [67]. Interestingly, LpR2 (Lipophorin Receptor 2), the fruit fly ortholog of AGAP002186, has been recently shown to suppress formation of melanotic tumors [68].

Reduction of the basal *LRIM1* promoter activity in cultured cells was detected only after silencing *REL2* compared to ds*LacZ*-treated control cells, consistent with an earlier study showing that REL2, as well as REL1, control basal *LRIM1* and *TEP1* expression [69].

### Perspective: a complex regulatory network of the hemocyte immune response

**Figure 5:**
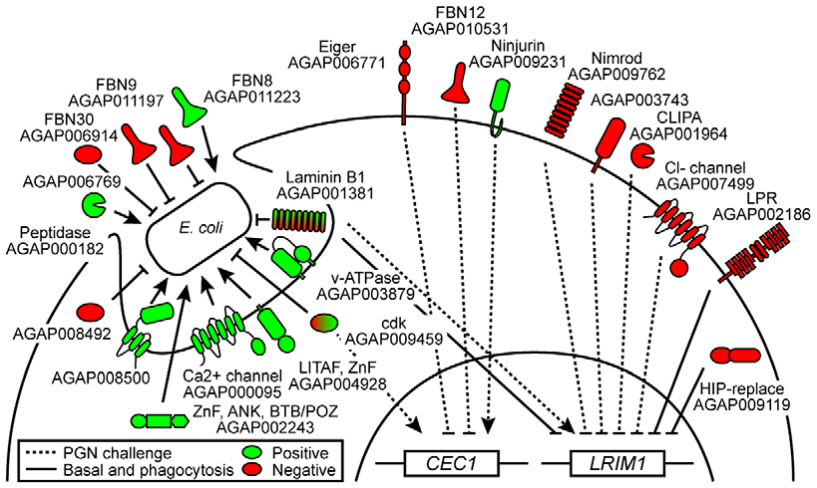
Schematic representation of the role of identified regulators in the *An. gambiae* hemocyte immune response. A total of 22 positive (green) and negative (red) regulators are shown. Dotted lines indicate modulations of *CEC1* and *LRIM1* gene expression upon PGN challenge, and solid lines describe the effect of genes on phagocytosis and basal expression of *LRIM1*.

Our results identify 22 novel regulators of the hemocyte immune response in this major African vector of human malaria. As summarized in Figure 5 and in Table S5, a complex network of positive and negative regulators of immediate (phagocytosis and basal complement state) as well as induced (AMP expression and induced complement state) responses are revealed.

Four membrane-bound or transmembrane proteins are implicated in *E. coli* phagocytosis, but none of them have domains that could indicate *bona fide* phagocytic receptors. Two of them, a putative Ca^2+^ channel protein (*AGAP000095*) and a v-ATPase (*AGAP003879*) have predicted functions that point to their involvement in phagosome assembly and maturation. Of the remaining two membrane-bound or transmembrane proteins, *AGAP004928* has an LPS-induced tumor necrosis factor alpha factor domain and could be involved in immune signaling [33], [34].

A very intriguing connection is revealed between phagocytosis and regulation of the basal and induced expression of LRIM1, a key component of the mosquito complement cascade [54], [55] and a known facilitator of *E. coli* phagocytosis [29]. The basement membrane protein Laminin B1 inhibits both phagocytosis and basal expression of LRIM1, but promotes LRIM1 expression after immune challenge. Therefore, Laminin B1 appears to play a dual role in maintaining the basal levels of complement in the hemolymph and in promoting production of complement components when needed or in preparation of potential reinfections. Whether this function of Laminin B1 is based on cell signaling and hemocyte differentiation, it remains to be investigated. Indeed, it has been suggested that immune priming of the mosquito hemolymph by gut bacteria following midgut invasion by malaria parasites causes hemocyte differentiation and attachment to the midgut basement membrane (basal lamina), which induces transient overexpression of LRIM1 and TEP1 [9].

Indeed complement is a very important defense reaction of the mosquito hemolymph [16], [29], [70]. Upon TEP1 maturation cleavage, TEP1_cut_ binds to the LRIM1/APL1C complex, where it remains in an active soluble state until an infection occurs [54], [55]. However, the last steps of TEP1 binding on the pathogen surface remain unknown. Rono and co-workers [67] have recently suggested that the observed antagonistic effect of Lp (and Vitellogenin) on TEP1 binding on malaria parasites may be due to Lp masking or competing for TEP1 binding sites on the parasite surface, directly interacting with TEP1, or modifying the lipid composition of the parasite membrane. Our finding that the Lp receptor suppresses the basal expression of *LRIM1* provides additional insights into the mechanism underlying the effect of Lp on TEP1-mediated parasite killing. Further investigation of the roles of the Lp receptor, Laminin B1 and the additional regulators of LRIM1 expression identified in this screen will shed light into the regulatory mechanisms of mosquito complement and how these impact upon infections with malaria parasites.

## Materials and Methods

### Mosquito rearing and dsRNA injections

*An. gambiae* G3 strain was maintained according to standard insectary procedures (www.mr4.org/). DsRNA injections in 2-days-old mosquitoes were performed as described previously [71].

### Cell cultures mainteinance and RNAi

The *An. gambiae* cell lines 4a2, 4a3A, 4a3B, L3-5, SuaE.1, SuaB.1, Sua4.0 and Sua5.1* were maintained as described (www.mr4.org/ and [12]). Briefly, cells were grown in Schneider's medium supplemented with 10% fetal bovine serum (heat inactivated), 100 U/ml Penicillin and 100 µg/ml Streptomycin at 27°C. Splitting was carried out by shaking flasks to detach cells and freezing/thawing procedures were performed according to standard cell culture protocols. RNAi-mediated gene silencing of cells was carried out in 96-well plates: approximately 10^5^ cultured cells were bathed in 1 µg dsRNA per well dissolved in serum-free Schneider's Medium and 2 h later complete medium was added to obtain a final serum concentration of 10%.

### RNA preparation, dsRNA synthesis and Quantitative RT-PCR

Total RNA from wild type, KD females and cultured cells was extracted using Trizol Reagent (Invitrogen). After DNAseI (Invitrogen) treatment, first strand cDNA was synthesized using oligo-d(T) primers (Invitrogen) and Superscript Reverse Transcriptase II (Invitrogen) according to the manufacturer's instructions.

For dsRNA synthesis, T7-tailed primers (see Table S1) were designed using the E-RNAi web-service at http://www.dkfz.de/signaling/e-rnai3/evaluation.php. PCR products were synthesized using cDNA from female mosquitoes as a template and purified using the QIAquick PCR Purification kit (QIAGEN). DsRNA synthesis was performed according to MEGAscript T7 Kit (Ambion) manufacturer's protocol and purification of dsRNA was performed by phenol/chloroform extraction. The quality and quantity of dsRNA were checked by agarose gel electrophoresis and Nanodrop reading, respectively.

Quantitative RT-PCR was performed using the SYBR Green PCR mastermix and analyzed using the ABI PRISM 7700 sequence detection system and the manufacturer's instructions. Expression levels were calculated by the relative standard curve method using S7 as endogenous control [72], [73]. Primers used are listed in Table S1.

### Viability assay

Cells were seeded in a 96-well plate at a concentration of 10^5^ cells/well. The next day the medium was removed and cells were treated with 1 µg dsRNA dissolved in 50 µl serum-free Schneider's Medium; 2 h later, complete medium was added to obtain a final serum concentration of 10% in a volume of 100 µl. Four days after dsRNA treatment, cells were stained with 1 µg/ml Hoechst 33342 (Invitrogen), and 500 nM Sytox Green (Invitrogen), by adding 25 µl of both chemicals to obtain a final volume of 150 µl/well. After 30 min incubation in the dark at 28°C, plates were analyzed by fluorescence microscopy. Images of cells were captured using a Zeiss Axiovert 200 widefield fluorescence microscope (10× objective) and the Improvision Volocity Software. Three images per well were taken (bright-field, DAPI and GFP). Images were analyzed using a protocol developed in ImageJ. Briefly, images were grouped in stacks and uniformly handled; stacks were transformed into binary images, and cells labeled with Hoechst and with Sytox Green were separately counted using Find Maxima Process Tool. Numbers obtained were statistically analyzed to quantify cell viability. ANOVA statistical analysis followed by Bonferroni's post-test were applied to groups of four to six pictures per dsRNA treatment.

To assess cell size, stacks were further processed to subtract background and enhance contrast and then transformed into binary images; cells were converted into particles, and their number, size and shape were calculated.

Viability assay was also performed using Cell Titer Blue kit (Promega). Four days after dsRNA treatment carried out as above, 20 µl/well of CellTiter Blue solution were added to obtain a final volume of 120 µl/well. Plates were incubated for 2 h and then fluorescence produced by reduction of substrate resazurin into resorufin was measured with BMG Labtech FLUOstar OMEGA plate reader. Three replicates were performed and z-score analysis was applied to identify positive phenotypes in each replicate. Thresholds of +/−2 were applied and positive candidates were considered those passing the threshold in at least two out of three replicates.

### Phagocytosis assay

Sua5.1* cells were seeded in 96-well plate at a concentration of 10^5^ cells/well. The next day the medium was removed and cells were treated with 1 µg dsRNA dissolved in 40 µl serum-free Schneider's Medium; 2 h later, complete medium was added to obtain a final serum concentration of 10% in a volume of 50 µl. PHRodo *E. coli* bioparticles (Invitrogen) were dissolved in sterile 1xPBS, sonicated according to manufacturer's instructions and 50 µl were added in each well 3 days after gene silencing to obtain a final volume of 100 µl/well. Cytochalasin D was added 20 min before bioparticle challenge at a concentration of 10 µM or 100 µM. BMG Labtech FLUOstar OMEGA plate reader was used to measure fluorescence intensity (Ex 532 nm/Em 595 nm filter) immediately after pHRodo bioparticles challenge, and at several time-points up to 24 h, keeping the temperature at 27°C during the entire procedure. Three replicates were performed. Plate reader outcomes were used to calculate z-score values for each replicate. Hits were considered as those dsRNAs producing z-score values above 2 or below −2 in at least two out of three replicates. Numerical values were also pooled and statistically analyzed using ANOVA followed by Tukey's Multiple Comparison Test to assess each dsRNA average value in relation to ds*LacZ*.

Automated fluorescence microscopy was also employed as a separate method to assess fluorescent pHRodo bioparticles uptake at 2 h and 20 h post challenge for those dsRNAs showing a phenotype according to the microplate reader assay. Images were captured using a Zeiss Axiovert 200 fluorescence widefield microscopy and the Volocity Improvision software (10× objective). Image analysis and quantification were performed using a protocol developed in ImageJ. Briefly, images were grouped in stacks and uniformly handled; background was subtracted, contrast enhanced and stacks were transformed into binary images to separate the fluorescent particles from the cell layer and quantify the spot number and size. Numbers obtained from at least two experiments were statistically analyzed using GraphPad Prism software. Numbers were converted to percent values compared to ds*LacZ*. After normality test, values of each dsRNA were compared to ds*LacZ* using student's t-test.

*In vivo* phagocytosis assays were performed on 2-day old mosquitoes injected with dsRNA as described above. Four days later, mosquitoes were injected again in the thorax with 69 nl of pHRodo *E. coli* bioparticles and allowed to recover and resume phagocytosis for 1 h. Mosquitoes were partially dissected (wings and legs removed) and gently compressed between a slide and a coverslip (using clay to hold them together) for imaging. Images of mosquito abdomens were captured using a Zeiss Axiovert 200 widefield fluorescence microscope and the Volocity Improvision software. At least two replicates for each dsRNA treatment were carried out, and at least 8 mosquitoes per KD were analyzed by microscopy with no less than four pictures captured per mosquito. Fluorescence images of sections of mosquito abdomens were captured as described above and shown in Figure 3 (10× objective). Quantification and statistical analysis were carried out as described above for pHRodo bioparticle phagocytosis in cultured cells.

### Luciferase assay

Sua5.1* cells were seeded in a 96-well plate at a concentration of 10^5^ cells per well. Upon reaching 80% confluence, the medium was removed and cells were treated with 1 µg dsRNA dissolved in 40 µl serum-free Schneider's Medium. Two h later, complete medium was added to obtain a final serum concentration of 10% in a volume of 60 µl. Four days after dsRNA treatment, cells were washed and co-transfected in 50 µl of final volume with *CEC1* or *LRIM1* promoter firefly luciferace fusion reporter constructs (Promega pGL3 backbone) and reference pAct5C-Renilla luciferase construct using Effectene Transfection Reagent (QIAGEN) following the manufacturer's instructions. One day after transfection, 25 µl of PGN (Sigma) were added to obtain a final concentration of 100 µg/ml. Seven h later, cells were subjected to Dual Luciferase assay (Promega) according to the manufacturer's instructions in a final volume of 150 µl per well. Relative light units per second for both firefly and renilla luciferase were measured using the Luminometer mode of BMG Labtech FLUOstar OMEGA plate reader. RLU measurements were obtained dividing the firefly by renilla measurements. RLUs from three replicates were averaged and z-scores calculated after PGN and PBS treatments. One-way ANOVA followed by Dunnet's multiple-comparison post-test was also performed to compare ds*LacZ* control and dsRNA RLUs.

### Data analysis

Z-score was calculated using the formula z_kj_ = (y_kj_−M)/S, where y_kj_ is the background subtracted value for the k^th^ well in the j^th^ replicate, and M and S are mean and standard deviation (SD) of the distribution of y values, respectively [74]. We considered positive hits those dsRNAs exhibiting z-scores>2 or <−2, which corresponds to SDs above or below the population mean in a given replicate. Considering that the critical z-score values when using a 95% confidence level are −1.96 and +1.96 SDs, positive hits have corresponding *p* values<0.05. To assess z-score correlation between replicates, D'Agostino & Pearson omnibus normality test was applied to examine whether the data follow a Gaussian distribution. Correlations were computed using Spearman r correlation test for not normally distributed data and Pearson r correlation test for normally distributed data. GraphPad Prism 5 software was used for statistical analyses and graph design.

## Supporting Information

Dataset S1 **dsRNA hemocyte-specific library.** The main features of dsRNAs and target genes used in this study are presented. DsRNA IDs, KD phenotypes in RNAi screens, VectorBase gene IDs, Affymetrix probe codes and previous ENSEMBL IDs are listed in columns A, B, C, D and E, respectively. Circulating hemocyte microarray information from [8] are summarized in columns F (cluster), G (normalized hemocyte value), H (normalized carcass value) and I (normalized head value). Comments (name and/or homology of *An. gambiae* genes) and IPRO domain data are reported in columns J and K, respectively. *D. melanogaster* orthologs are shown in column L, and corresponding FlyBase IDs in column M. *D. melanogaster* KD phenotypes according to the GenomeRNAi database are reported in column N. For further details see Text S1.
(XLS)

Figure S1 **Quantitative analysis of viability assays.** (A) Images of L3–5 confluent cell layers after dsRNA treatments as indicated on the left side of each figure. Nuclei are stained with Hoechst, and dead cells are stained with Sytox Green. (B) Viability assay analysis of 8 *An. gambiae* cell lines treated with dsRNAs targeting *IAP1*, *AGAP005160* and *AGAP008001*. Total number of cells (Hoechst) and dead cells (Sytox Green) were counted by image analysis using a protocol developed in ImageJ. Ratios of dead and total number of cells are reported in the graphs. Three pictures taken from two independent replicates were analyzed, and averages and standard errors are shown. One-way ANOVA followed by Bonferroni's Multiple Comparison Test was applied (*, p<0,05; **, p<0,01; ***, p<0,001). (C) Kaplan-Meier survival curves of adult mosquitoes following injection with dsRNA targeting *IAP1* and *AGAP008001*. Percent survival compared to ds*LacZ* treated controls is shown.
(TIF)

Figure S2 **Qualitative analysis of viability assays.** (A) Nuclear staining with Hoechst of Sua4.0 cultured cells that are either untreated (no dsRNA) or treated with dsRNA targeting *LacZ* (ds*LacZ*) and *AGAP005160* (ds*5160*). Note that after ds*5160* treatment, cells showed impaired cytokinesis and increased nuclear size. (B) Quantification of the number of cells by counts of Hoechst positive nuclei and (C) measurements of nuclear size, using a protocol developed in ImageJ. Pictures taken from three independent replicates were analyzed. Mean values and standard errors are shown. One-way ANOVA followed by Tukey's Multiple Comparison Test was used for statistical analyses (*, p<0,05; ***, p<0,001).
(TIF)

Figure S3 **Z-score correlation between replicates in viability assays.** D'Agostino & Pearson omnibus normality test revealed that data do not fit a normal distribution. Therefore, correlation between z-score values of replicates I, II and III was calculated using Spearman r correlation test. Correlation coefficients (r) are indicated. Positive hits (z-scores>2 or <−2 in at least two of three replicates) are shown as open circles. Control no dsRNA-treated samples and samples treated with 100 mM H_2_O_2_ are also shown.
(TIF)

Figure S4 **Phagocytosis assay.** (A) Sua5.1* cells treated with ds*LacZ*, ds*Cactus* and ds*BINT2* were challenged with *E. coli* and *Staphylococcus aureus* pHRodo bioparticles and the levels of phagocytosis were measured 1 h later by microtiter plate reading. Values were background subtracted. (B) Sua5.1* cells were treated with Cytochalasin D at concentrations of 10 mM and 100 mM in conditioned medium and then challenged with pHRodo *E. coli*. The levels of phagocytosis were measured at different time points (TP) as indicated. Values were background subtracted. (C) Z-score plots relative to microplate reader measurements of *E. coli* pHRodo conjugated bioparticles uptake at different TPs. Each dsRNA was screened in triplicate and positive hits were considered as those dsRNAs with z-score in at least 2 out of the 3 replicates >2 (red) or <−2 (green). 0 h TP (immediately after bioparticle challenge) was subtracted in the graphs TP 1-0, TP 3-0, TP 6-0 and TP 24-0 (measurements at 1 h, 3 h, 6 h and 24 h, respectively). Z-scores at TP 1, 3 and 6 (1, 3 and 6 h after challenge, without subtracting the TP 0 h, respectively) are shown, as well as z-score plots indicating the kinetic of the uptake between 1 h and 3 h after challenge (TP 3-1). (D) Correlation between z-score values of different replicates. TPs selected are indicated on top of each graph, and replicates compared are specified at the left of each graph. Correlation coefficients (r) are also indicated.
(TIF)

Figure S5 **Gene-specific KD efficiency in cell cultures (A–D) and mosquitoes (E).** Confluent Sua5.1* cells were incubated with dsRNAs targeting AGAP004016 (A), AGAP004928 (B), AGAP005227 (C) and AGAP009201 (D), and 4 days later the expression of targeted genes was analyzed by qRT-PCR. Data were normalized to *S7* and calibrated to the gene-specific expression in ds*LacZ* treated samples. Three wells for each KD were analyzed. The level of silencing of three of these genes in mosquitoes was also tested (E). dsRNAs targeting AGAP004928, AGAP005227, AGAP009201 and against *LacZ* were injected into 2-day old female mosquitoes and the expression of silenced genes was measured by qRT-PCR 4 days after dsRNA treatments. Data were normalized to *S7* levels and calibrated to the gene-specific expression in ds*LacZ* treated mosquitoes.
(TIF)

Table S1 **List of primers used in this study.** Name of dsRNA (#), AGAP code (ID), T7-tailed primers code (Primer Forward and Primer Reverse), and relative sequences are reported. Primers for Q-PCR (QF and QR) are listed at the bottom of the table.
(DOC)

Table S2 **Genes exploited as controls in viability assays.** Name of dsRNA (#), AGAP code (IDs), gene name, IPRO ID and length of T7 dsRNA products are reported.
(DOC)

Table S3 **Phagocytosis assay results.** Datasets from *in vitro* measurements using microplate reader were statistically analysed employing two statistical approaches, z-score threshold and ANOVA calculation. In column “#” are listed dsRNA labels; AGAP ID number and IPRO domain descriptions are reported in the next two columns. “Z-score” column lists genes with significant values at the indicated TP (for at least two replicates out of three) from plate reader measurements. Microplate reader values were also averaged and compared to dsLacZ control values: positive hits for each TP, according to ANOVA statistical analysis followed by Tukey's Multiple Comparison Test, are listed in “ANOVA P<0,05” column. 8 genes selected from these 13 significant candidates were evaluated in *in vivo* assay. The “*in vivo* % TP2” column reports the percent of phagocytosis as calculated by imaging analysis in *in vivo* experiments 2 h after challenge. SP, Signal Peptide; TD, transmembrane domain; ns, not significant; nd, not determined.
(DOC)

Table S4 **Luciferase assay results.** Gene knockdowns (KD) that modulate the regulation of CEC and LRIM1 promoters upon PGN challenge according to the z-score analysis are shown; KDs modulating basal LRIM1 promoter activity (PBS challenge) are also summarized; IPRO domains short descriptions are reported. SP, Signal peptide; TD, transmembrane domain.
(DOC)

Table S5 **Summary of RNAi screens results.** In column “#” are listed dsRNA labels; AGAP ID number and IPRO domain descriptions and homologies are reported in the next 2 columns. KD phenotypes of genes that gave a positive phenotype in at least one of the 4 assays are summarized in the next 4 columns.
(DOC)

Text S1 **Additional information and data on viability assay,** ***ex vivo*** **phagocytosis assay, knockdown efficiency assessment and** ***Drosophila melanogaster*** **orthologs.**
(DOC)
