# Supplementary figures and images for: Comprehensive Genetic Dissection of the Hemocyte Immune Response in the Malaria Mosquito Anopheles gambiae
Source: PLoS Pathog. 2013 Jan 31;9(1):e1003145. doi: 10.1371/journal.ppat.1003145 (PMC3561300; doi:10.1371/journal.ppat.1003145)

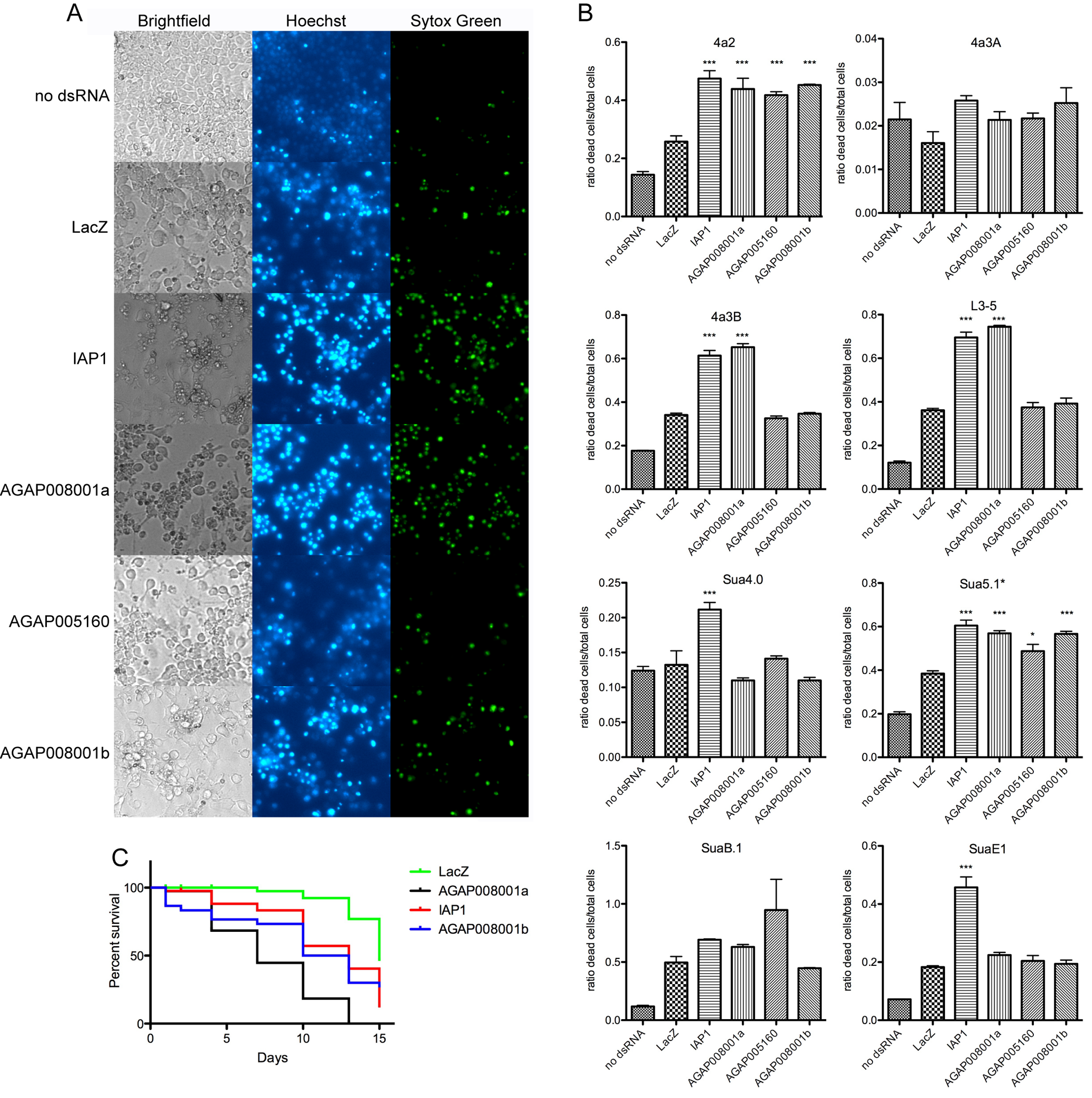

Supplement: Figure S1 — Quantitative analysis of viability assays. (A) Images of L3–5 confluent cell layers after dsRNA treatments as indicated on the left side of each figure. Nuclei are stained with Hoechst, and dead cells are stained with Sytox Green. (B) Viability assay analysis of 8 An. gambiae cell lines treated with dsRNAs targeting IAP1, AGAP005160 and AGAP008001. Total number of cells (Hoechst) and dead cells (Sytox Green) were counted by image analysis using a protocol developed in ImageJ. Ratios of dead and total number of cells are reported in the graphs. Three pictures taken from two independent replicates were analyzed, and averages and standard errors are shown. One-way ANOVA followed by Bonferroni's Multiple Comparison Test was applied (*, p<0,05; **, p<0,01; ***, p<0,001). (C) Kaplan-Meier survival curves of adult mosquitoes following injection with dsRNA targeting IAP1 and AGAP008001. Percent survival compared to dsLacZ treated controls is shown. (TIF) [file ppat.1003145.s002.tif]

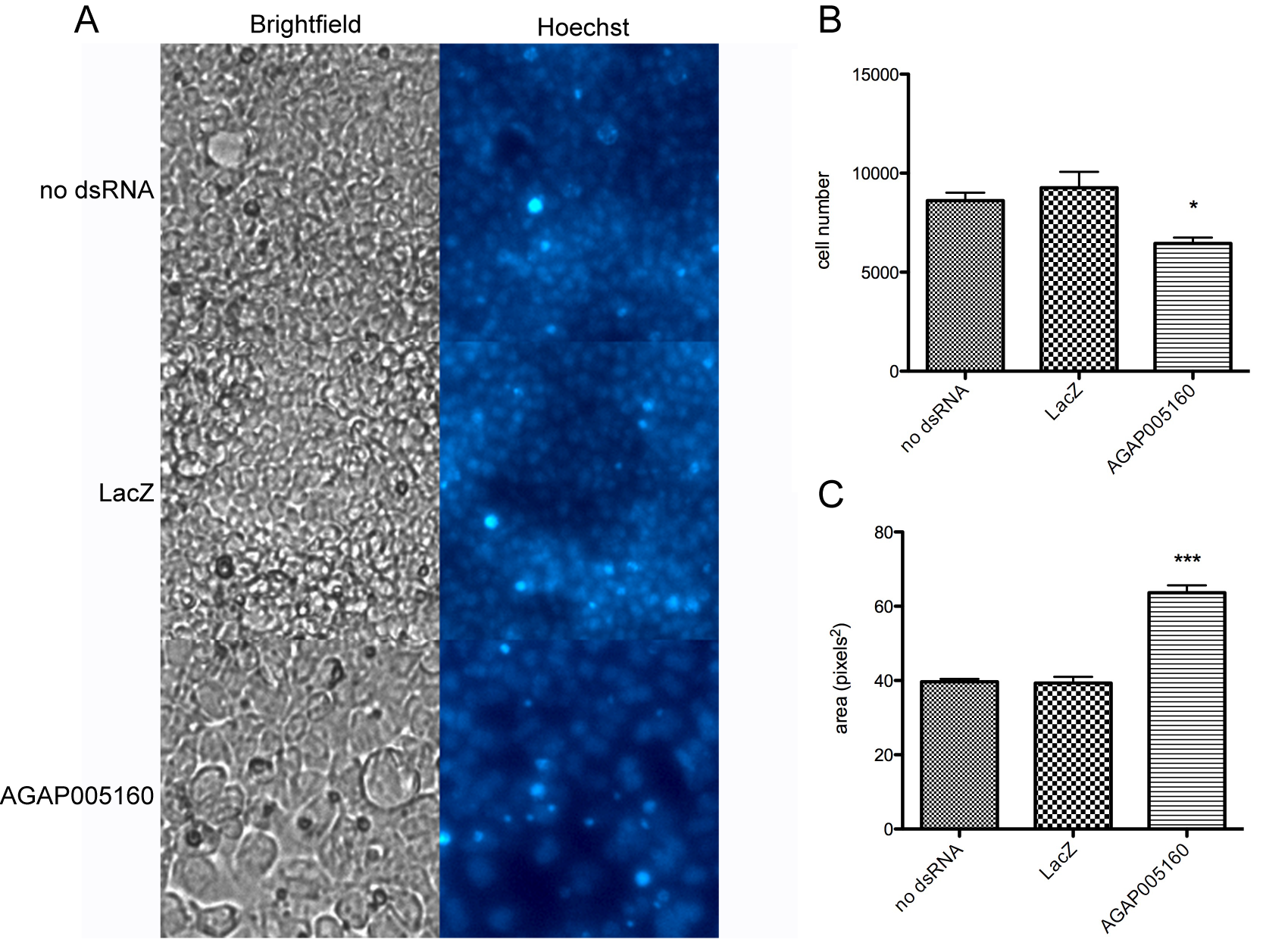

Supplement: Figure S2 — Qualitative analysis of viability assays. (A) Nuclear staining with Hoechst of Sua4.0 cultured cells that are either untreated (no dsRNA) or treated with dsRNA targeting LacZ (dsLacZ) and AGAP005160 (ds5160). Note that after ds5160 treatment, cells showed impaired cytokinesis and increased nuclear size. (B) Quantification of the number of cells by counts of Hoechst positive nuclei and (C) measurements of nuclear size, using a protocol developed in ImageJ. Pictures taken from three independent replicates were analyzed. Mean values and standard errors are shown. One-way ANOVA followed by Tukey's Multiple Comparison Test was used for statistical analyses (*, p<0,05; ***, p<0,001). (TIF) [file ppat.1003145.s003.tif]

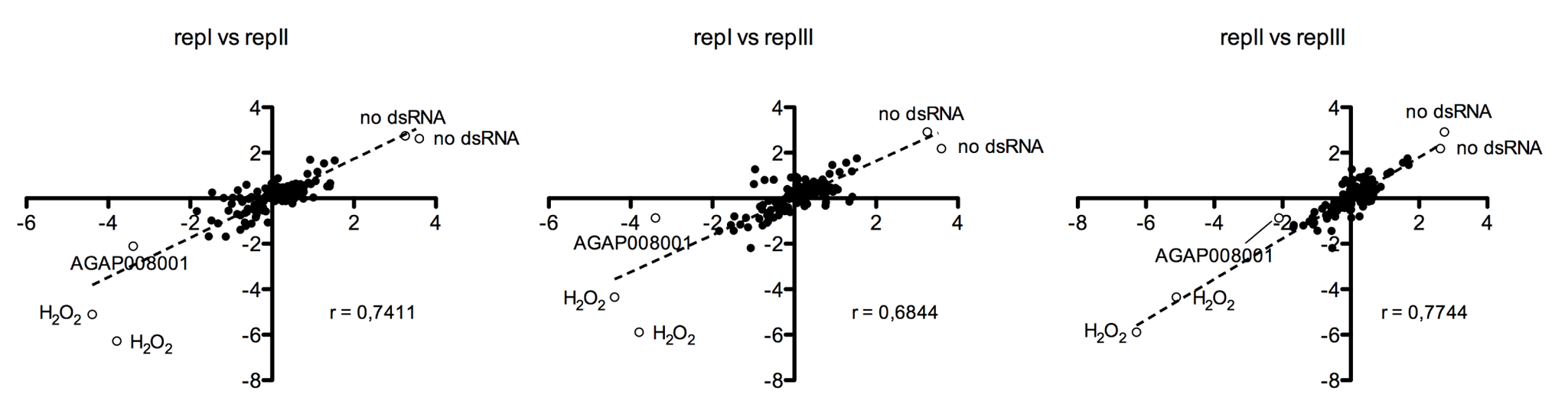

Supplement: Figure S3 — Z-score correlation between replicates in viability assays. D'Agostino & Pearson omnibus normality test revealed that data do not fit a normal distribution. Therefore, correlation between z-score values of replicates I, II and III was calculated using Spearman r correlation test. Correlation coefficients (r) are indicated. Positive hits (z-scores>2 or <−2 in at least two of three replicates) are shown as open circles. Control no dsRNA-treated samples and samples treated with 100 mM H2O2 are also shown. (TIF) [file ppat.1003145.s004.tif]

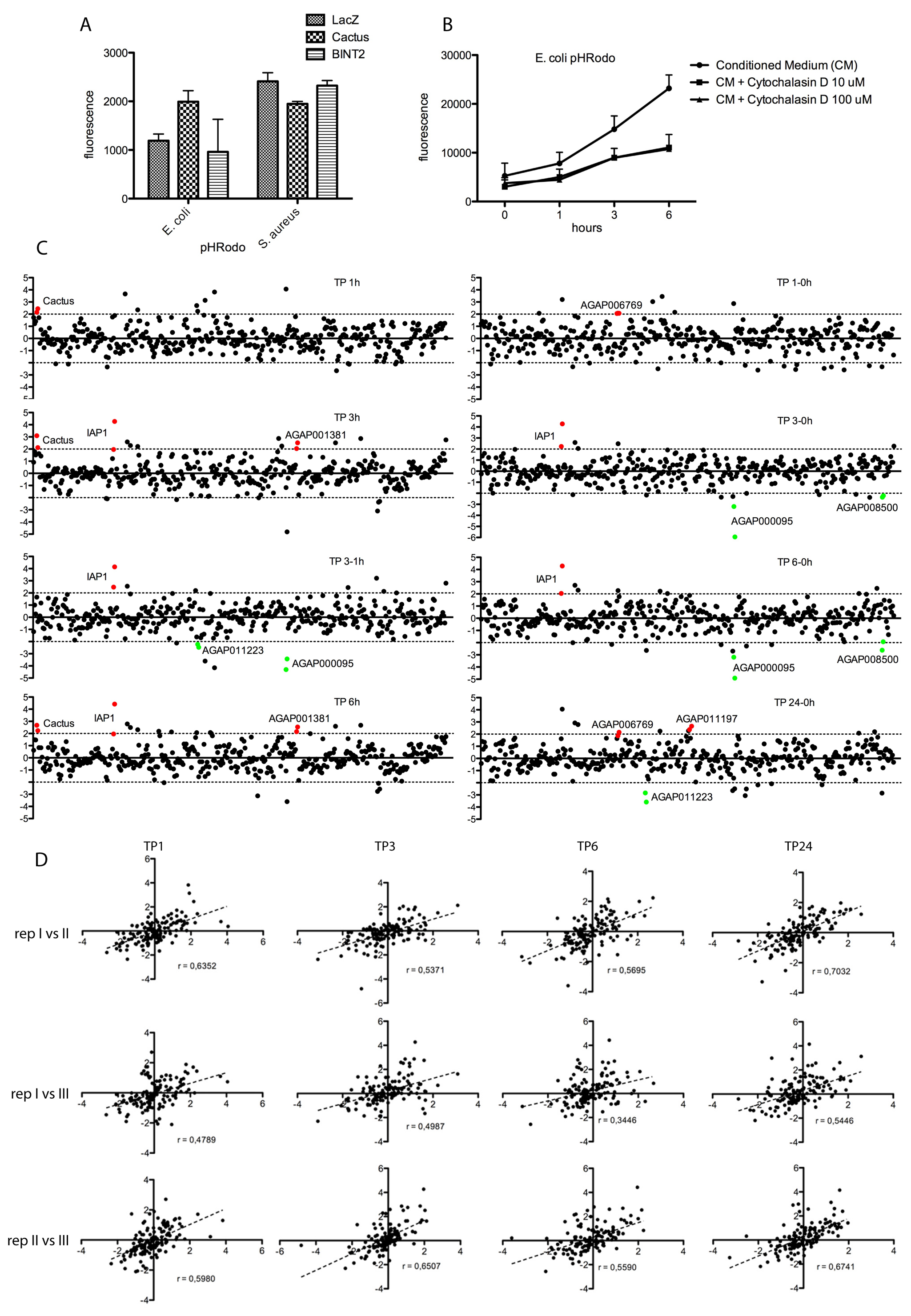

Supplement: Figure S4 — Phagocytosis assay. (A) Sua5.1* cells treated with dsLacZ, dsCactus and dsBINT2 were challenged with E. coli and Staphylococcus aureus pHRodo bioparticles and the levels of phagocytosis were measured 1 h later by microtiter plate reading. Values were background subtracted. (B) Sua5.1* cells were treated with Cytochalasin D at concentrations of 10 mM and 100 mM in conditioned medium and then challenged with pHRodo E. coli. The levels of phagocytosis were measured at different time points (TP) as indicated. Values were background subtracted. (C) Z-score plots relative to microplate reader measurements of E. coli pHRodo conjugated bioparticles uptake at different TPs. Each dsRNA was screened in triplicate and positive hits were considered as those dsRNAs with z-score in at least 2 out of the 3 replicates >2 (red) or <−2 (green). 0 h TP (immediately after bioparticle challenge) was subtracted in the graphs TP 1-0, TP 3-0, TP 6-0 and TP 24-0 (measurements at 1 h, 3 h, 6 h and 24 h, respectively). Z-scores at TP 1, 3 and 6 (1, 3 and 6 h after challenge, without subtracting the TP 0 h, respectively) are shown, as well as z-score plots indicating the kinetic of the uptake between 1 h and 3 h after challenge (TP 3-1). (D) Correlation between z-score values of different replicates. TPs selected are indicated on top of each graph, and replicates compared are specified at the left of each graph. Correlation coefficients (r) are also indicated. (TIF) [file ppat.1003145.s005.tif]

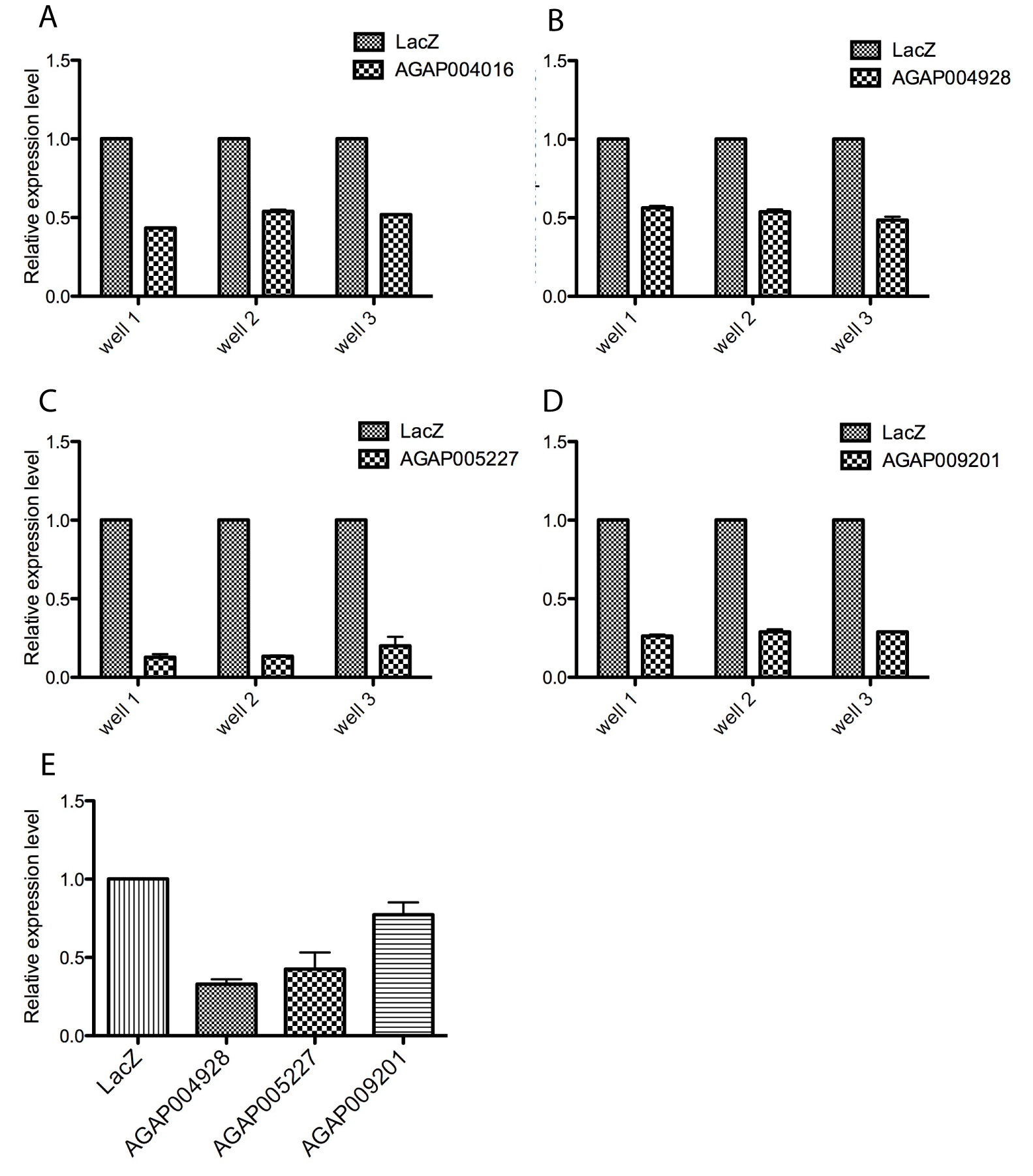

Supplement: Figure S5 — Gene-specific KD efficiency in cell cultures (A–D) and mosquitoes (E). Confluent Sua5.1* cells were incubated with dsRNAs targeting AGAP004016 (A), AGAP004928 (B), AGAP005227 (C) and AGAP009201 (D), and 4 days later the expression of targeted genes was analyzed by qRT-PCR. Data were normalized to S7 and calibrated to the gene-specific expression in dsLacZ treated samples. Three wells for each KD were analyzed. The level of silencing of three of these genes in mosquitoes was also tested (E). dsRNAs targeting AGAP004928, AGAP005227, AGAP009201 and against LacZ were injected into 2-day old female mosquitoes and the expression of silenced genes was measured by qRT-PCR 4 days after dsRNA treatments. Data were normalized to S7 levels and calibrated to the gene-specific expression in dsLacZ treated mosquitoes. (TIF) [file ppat.1003145.s006.tif]
