# Supplementary material for: Comprehensive Genetic Dissection of the Hemocyte Immune Response in the Malaria Mosquito Anopheles gambiae
Source: PLoS Pathog. 2013 Jan 31;9(1):e1003145. doi: 10.1371/journal.ppat.1003145 (PMC3561300; doi:10.1371/journal.ppat.1003145)
Supplement: Table S1 — List of primers used in this study. Name of dsRNA (#), AGAP code (ID), T7-tailed primers code (Primer Forward and Primer Reverse), and relative sequences are reported. Primers for Q-PCR (QF and QR) are listed at the bottom of the table. (DOC) [file ppat.1003145.s007.doc]

**Table S1.** List of primers used in this study.

| *#* | *ID* | *Primer Forward* | *Sequence* | *Primer Reverse* | *Sequence* |
| --- | --- | --- | --- | --- | --- |
| A1 | AGAP000182 | AGAP000182_F | TAATACGACTCACTATAGGGCAGCATATATGTGCGGGTTG | AGAP000182_R | TAATACGACTCACTATAGGGCGACGAAAAGATGGTTGAT |
| A2 | AGAP000220 | AGAP000220_F | TAATACGACTCACTATAGGGCAGCGGTACAACTTCAACA | AGAP000220_R | TAATACGACTCACTATAGGGCAGGCTGGAGTTTTTGTTCC |
| A3 | AGAP000249 | AGAP000249_F | TAATACGACTCACTATAGGGATCTGATCGTGTGCTACGC | AGAP000249_R | TAATACGACTCACTATAGGGCTCTTTGTGCAGCTTGATG |
| A4 | AGAP000305 | AGAP000305_F | TAATACGACTCACTATAGGGAGCTGGGTGAGAGATGTTGG | AGAP000305_R | TAATACGACTCACTATAGGGTCAGATCGTCCTCCTCCAG |
| A5 | AGAP000385 | AGAP000385_F | TAATACGACTCACTATAGGGACATCTGTTCTTCCGCGTCT | AGAP000385_R | TAATACGACTCACTATAGGGTCCTGCTCGGAGAGAGAGAG |
| A6 | AGAP001954 | AGAP001954_F | TAATACGACTCACTATAGGGACAAGCTGCACGATAAT | AGAP001954_R | TAATACGACTCACTATAGGGTCCACAAGTCCAGCACAAAG |
| A7 | AGAP002243 | AGAP002243_F | TAATACGACTCACTATAGGGCGAACATGCAAAACAGCAAG | AGAP002243_R | TAATACGACTCACTATAGGGTGCAGTGATCGGTCAGAAAG |
| A8 | AGAP002415 | AGAP002415_F | TAATACGACTCACTATAGGGTGTCGTTATGCTCGAACAGC | AGAP002415_R | TAATACGACTCACTATAGGGCAGCGTTTCGTTGAAGATGA |
| A9 | TCLAG163691 | AGAP003878_F | TAATACGACTCACTATAGGGAAGCTCTATCTGGCCCACAA | AGAP003878_R | TAATACGACTCACTATAGGGCAGAACATCTCTGGGTGGT |
| A10 | AGAP003879 | AGAP003879_F | TAATACGACTCACTATAGGGCCTTGGTTGAGGCACAGATT | AGAP003879_R | TAATACGACTCACTATAGGGTACGAGCCGATGATGATGAA |
| A11 | AGAP003898 | AGAP003898_F | TAATACGACTCACTATAGGGTTCCTGCCCGACTTTATCAC | AGAP003898_R | TAATACGACTCACTATAGGGTTTTGCTCCAGCGTCTCTC |
| A12 | AGAP003960 | AGAP003960_F | TAATACGACTCACTATAGGGCCCACTGTTTCAGTAACG | AGAP003960_R | TAATACGACTCACTATAGGGCGCAGCGGGTAGTAGATAG |
| A13 | AGAP004017 | AGAP004017_F | TAATACGACTCACTATAGGGCGCATTCCTGCAGCATACTA | AGAP004017_R | TAATACGACTCACTATAGGGCCAGGTACCGTAAGGCGTAA |
| A14 | AGAP004928 | AGAP004928_F | TAATACGACTCACTATAGGGCCTCCGACAATAAGCGTCAT | AGAP004928_R | TAATACGACTCACTATAGGGCCGTCCCAAATATGCTTTA |
| A15 | AGAP004993B | AGAP004993B_F | TAATACGACTCACTATAGGGCTTGGAGCTTGAAGGTGGAG | AGAP004993B_R | TAATACGACTCACTATAGGGTGAGCGTAGCGTTACGAATG |
| A16 | AGAP004993C | AGAP004993C_F | TAATACGACTCACTATAGGGCAAGCTGGTAGTGGATA | AGAP004993C_R | TAATACGACTCACTATAGGGTGATCATCTCCAGCACGAAG |
| A17 | AGAP005174 | AGAP005174_F | TAATACGACTCACTATAGGGCTCAAACCAATCCTCGATCC | AGAP005174_R | TAATACGACTCACTATAGGGTGATTGTGCCTCGCTTGTAG |
| A18 | AGAP005227 | AGAP005227_F | TAATACGACTCACTATAGGGCCGAGTTCCAGATTGACGAT | AGAP005227_R | TAATACGACTCACTATAGGGCAGGCATTCCTTGGACATTT |
| A19 | AGAP005549 | AGAP005549_F | TAATACGACTCACTATAGGGATGACGAGTTCGATGCGTA | AGAP005549_R | TAATACGACTCACTATAGGGAGACAATCGGTTGGATCGAG |
| A20 | AGAP005551 | AGAP005551_F | TAATACGACTCACTATAGGGATTGCGCTGAACGAAGAGAT | AGAP005551_R | TAATACGACTCACTATAGGGTCGCACTCATCGTACCGTAG |
| A21 | AGAP005992 | AGAP005992_F | TAATACGACTCACTATAGGGTCGCTCATGGAGGAGTACCT | AGAP005992_R | TAATACGACTCACTATAGGGATGCACTGGTTCCTTTGT |
| A22 | AGAP006769 | AGAP006769_F | TAATACGACTCACTATAGGGTACACACTGCACGCACGTAA | AGAP006769_R | TAATACGACTCACTATAGGGCTCTTCTGGTGCGGAATCTC |
| A23 | AGAP007499 | AGAP007499_F | TAATACGACTCACTATAGGGACGTCGACAGTAGCGATGA | AGAP007499_R | TAATACGACTCACTATAGGGTTCAGCAGCGACCAGTACA |
| A24 | AGAP007540 | AGAP007540_F | TAATACGACTCACTATAGGGTACCATCAGCTGGCCATACA | AGAP007540_R | TAATACGACTCACTATAGGGACACATGAACGACAGCTTGC |
| A25 | AGAP008086 | AGAP008086_F | TAATACGACTCACTATAGGGCTGAACCGGTACCACTGGAT | AGAP008086_R | TAATACGACTCACTATAGGGTGTCGCTTGGAGAAGGCTAT |
| A26 | AGAP008492 | AGAP008492_F | TAATACGACTCACTATAGGGTTCTGCAGCAACATCTGACC | AGAP008492_R | TAATACGACTCACTATAGGGAAACGTTGCGATATCCTTGG |
| A27 | AGAP008521 | AGAP008521_F | TAATACGACTCACTATAGGGTCGATCTCACCGAAGGGTA | AGAP008521_R | TAATACGACTCACTATAGGGCCGTCAGCTCGGAGTTAAAA |
| A28 | AGAP008908 | AGAP008908_F | TAATACGACTCACTATAGGGTCATCAAGGACATCGAGCAG | AGAP008908_R | TAATACGACTCACTATAGGGCAGGTTGGTGTCCGAGTTTT |
| A29 | AGAP008909 | AGAP008909_F | TAATACGACTCACTATAGGGAGCATGGAGAAGCACGAAGT | AGAP008909_R | TAATACGACTCACTATAGGGAGCTGGTACTTCTCGGCAAA |
| A30 | AGAP009119 | AGAP009119_F | TAATACGACTCACTATAGGGCTGGAAATTAACCCCGACT | AGAP009119_R | TAATACGACTCACTATAGGGTCATCAGCACCGACATGATT |
| A31 | AGAP009200 | AGAP009200_F | TAATACGACTCACTATAGGGACTATCCGCACAACCAGGAC | AGAP009200_R | TAATACGACTCACTATAGGGAACCAGCCAGAAGGAGGTTT |
| A32 | AGAP009201 | AGAP009201_F | TAATACGACTCACTATAGGGATGCCGTTCATGTTCTGTGA | AGAP009201_R | TAATACGACTCACTATAGGGTCAATGATCGTCAGCCAGA |
| A33 | AGAP009459 | AGAP009459_F | TAATACGACTCACTATAGGGAGATCGTGACGCTGTGGTA | AGAP009459_R | TAATACGACTCACTATAGGGAAGGAATGGCAAAACACCTG |
| A34 | AGAP009792 | AGAP009792_F | TAATACGACTCACTATAGGGTACGTGACGGACGATGATG | AGAP009792_R | TAATACGACTCACTATAGGGTCGACGAGCTGAATGATCTG |
| A35 | AGAP010580 | AGAP010580_F | TAATACGACTCACTATAGGGCAGGGTGTTGTCCTCCTGTT | AGAP010580_R | TAATACGACTCACTATAGGGCTTCTTGGGCATCATCGTTT |
| A36 | AGAP010587 | AGAP010587_F | TAATACGACTCACTATAGGGCAATGGAATGAAGCAAAGCA | AGAP010587_R | TAATACGACTCACTATAGGGTAGGAGATGAGCGTGAGC |
| A37 | AGAP011223 | AGAP011223_F | TAATACGACTCACTATAGGGTGGCTAGTGATTCAGCATCG | AGAP011223_R | TAATACGACTCACTATAGGGTGCCAATCCCTGGTAAGAAG |
| A38 | AGAP011984 | AGAP011984_F | TAATACGACTCACTATAGGGCGGTGGTAAGCGATATGAT | AGAP011984_R | TAATACGACTCACTATAGGGCGGTACTGGAATGTGTCGTG |
| A39 | AGAP012034 | AGAP012034_F | TAATACGACTCACTATAGGGTCATGCCCGACTGAATGATA | AGAP012034_R | TAATACGACTCACTATAGGGTGTACTCTGACACCCGCATC |
| A40 | AGAP012614 | AGAP012614_F | TAATACGACTCACTATAGGGACCCCTCACGCTTTATGTTG | AGAP012614_R | TAATACGACTCACTATAGGGAGTTGCCAAATCCCACTCAC |
| 1 | AGAP000790 | ENST21342_F | TAATACGACTCACTATAGGGTCCCACACACACACAACCCCAG | ENST21342_R | TAATACGACTCACTATAGGGACAGTGCATGATGAGGGCCCAG |
| 2 | AGAP004016 | ENST14345_F | TAATACGACTCACTATAGGGTGCTTCACGAGATCCTAAAG | ENST14345_R | TAATACGACTCACTATAGGGAATGTTCAAGCTGTTCAGGAAA |
| 3 | AGAP012000 | ENST16460_F | TAATACGACTCACTATAGGGATACAAGAAACTAACATCATGTTCA | ENST16460_R | TAATACGACTCACTATAGGGTACTGGCCAACCGATTTAATC |
| 4 | AGAP006743 | ENST20316_F | TAATACGACTCACTATAGGGTCCCTTCAAGGAACCGATCACG | ENST20316_R | TAATACGACTCACTATAGGGTCACCGCGCAAATACTTTCCG |
| 5 | AGAP001508 | ENST09630_F | TAATACGACTCACTATAGGGCGTGTCTGTTGGTGCTGGTTGC | ENST09630_R | TAATACGACTCACTATAGGGACCTACGTTCGTGCGGAAATGG |
| 6 | AGAP009762 | ENST14402_F | TAATACGACTCACTATAGGGCATCCCACCGATGGGCATT | ENST14402_R | TAATACGACTCACTATAGGGTCCCTCGACAAACACAAATCC |
| 7 | AGAP009387 | ENST26294_F | TAATACGACTCACTATAGGGATTCCCGCGCACTGGTTTTG | ENST26294_R | TAATACGACTCACTATAGGGTGGCTCCAGTCGGTAACGTTCA |
| 8 | AGAP006771 | ENST20363_F | TAATACGACTCACTATAGGGTTCGACGGGCATGATGAGTACG | ENST20363_R | TAATACGACTCACTATAGGGCGATTTTCTACATCCCAGCGG |
| 9 | AGAP000376 | ENST21949_F | TAATACGACTCACTATAGGGAGGGTGCGATCCGCTG | ENST21949_R | TAATACGACTCACTATAGGGCAGCCGCAGCACGTTC |
| 10 | AGAP006914 | ENST21698_F | TAATACGACTCACTATAGGGCTCAAAATCAATCCCAACGG | ENST21698_R | TAATACGACTCACTATAGGGTCGTTATCCTGATCGTAGGT |
| 11 | AGAP011197 | ENST11248_F | TAATACGACTCACTATAGGGAGAAGTCTACGGATCCGAC | ENST11248_R | TAATACGACTCACTATAGGGCTTGGTCTGCTCGCAGTAC |
| 12 | AGAP007312 | ENST16759_F | TAATACGACTCACTATAGGGTGCCGATCTCCAGCGCCTTA | ENST16759_R | TAATACGACTCACTATAGGGATAGAGCAGCGCGAACGGCA |
| 13 | AGAP009231 | ENST10584_F | TAATACGACTCACTATAGGGTGCTCGGCACGCAAACGTA | ENST10584_R | TAATACGACTCACTATAGGGCGGATCGTTCACCTGCGTGA |
| 14 | AGAP002594 | ENST10605_F | TAATACGACTCACTATAGGGCTTGTTTGATGATCGCGATGGC | ENST10605_R | TAATACGACTCACTATAGGGTCGACCACATTCTTCGCGAGTG |
| 15 | AGAP001662 | ENST22061_F | TAATACGACTCACTATAGGGCGGAAACGTGCAAGGAG | ENST22061_R | TAATACGACTCACTATAGGGTGCGCCGGCGTTTTTC |
| 16 | AGAP001243 | ENST23056_F | TAATACGACTCACTATAGGGAGCAAGACGGAAGCACGAAACC | ENST23056_R | TAATACGACTCACTATAGGGTGCTTCAGTTTCAAGGTGGCCG |
| 17 | AGAP012337 | ENST28160_F | TAATACGACTCACTATAGGGACTACACCGGCTGCGTGTTTG | ENST28160_R | TAATACGACTCACTATAGGGCAGATATCGATCGGCAGGTCC |
| 18 | AGAP009380 | ENST25171_F | TAATACGACTCACTATAGGGACGAATCGCGATCAGCACGG | ENST25171_R | TAATACGACTCACTATAGGGAAGTCGAGATGCTGTTCCGCG |
| 19 | AGAP007039 | ENST15066_F | TAATACGACTCACTATAGGGCCACGATTTACGTTACCAAG | ENST15066_R | TAATACGACTCACTATAGGGAGCCGCGGCCAGTAG |
| 20 | AGAP011765 | ENST08856_F | TAATACGACTCACTATAGGGAGCGGACTCGACCTGTG | ENST08856_R | TAATACGACTCACTATAGGGCTCCGCAACGCACATCTC |
| 21 | AGAP008013 | ENST12392_F | TAATACGACTCACTATAGGGTTCCGATCGTAGTACAGCCCGC | ENST12392_R | TAATACGACTCACTATAGGGTCTGAGGCCCACTTTGAGCTGC |
| 22 | AGAP009556 | ENST29396_F | TAATACGACTCACTATAGGGCTCACTGCCACACGGTTGAAGG | ENST29396_R | TAATACGACTCACTATAGGGAAAGTGATCCCGGCCGTTGTC |
| 23 | AGAP012386 | ENST08964_F | TAATACGACTCACTATAGGGTGTCAAAATGGAACCTGTACCC | ENST08964_R | TAATACGACTCACTATAGGGCATTCGCAAACATTCACACT |
| 24 | AGAP011668 | ENST28145_F | TAATACGACTCACTATAGGGAGTGATTGACAACCCTTC | ENST28145_R | TAATACGACTCACTATAGGGCGTAACCAAACAACTAC |
| 25 | AGAP000095 | ENST08160_F | TAATACGACTCACTATAGGGACGATAGCGGCCGTCTT | ENST08160_R | TAATACGACTCACTATAGGGCCGGAACTCCTTGAACTTCT |
| 26 | AGAP005612 | ENST18284_F | TAATACGACTCACTATAGGGTTTCGGCACTGGTGATCGGTG | ENST18284_R | TAATACGACTCACTATAGGGCACGTCCTTGTTGCCGGGAA |
| 27 | AGAP000806 | ENST21380_F | TAATACGACTCACTATAGGGTGGGAGAAGTACCGCGAC | ENST21380_R | TAATACGACTCACTATAGGGCGGCGCTTCTTTATCTC |
| 28 | AGAP001381 | ENST16875_F | TAATACGACTCACTATAGGGCCGGTATACTCGCGCAAC | ENST16875_R | TAATACGACTCACTATAGGGATCTCGTACGGCGAGTC |
| 29 | AGAP009490 | ENST25538_F | TAATACGACTCACTATAGGGCACGACCGTCGCAATCTCAATC | ENST25538_R | TAATACGACTCACTATAGGGCCTGCTTGATGAACAGCTCGA |
| 30 | AGAP006327 | ENST13779_F | TAATACGACTCACTATAGGGATCGGGGGTGATTCGATACA | ENST13779_R | TAATACGACTCACTATAGGGCAGCGAGAGCGTCTGC |
| 31 | AGAP010531 | ENST20573_F | TAATACGACTCACTATAGGGCTCGAGCAAGAACGTCTGA | ENST20573_R | TAATACGACTCACTATAGGGTAAGGTAGTAGCATCTTGGTCA |
| 32 | AGAP012352 | ENST20083_F | TAATACGACTCACTATAGGGCCGAGTTTGCCGCAAACGA | ENST20083_R | TAATACGACTCACTATAGGGTTATTGACCGCCGTTGCGC |
| 33 | AGAP005611 | ENST17826_F | TAATACGACTCACTATAGGGATGGCGCGTGTTTCGAGCAG | ENST17826_R | TAATACGACTCACTATAGGGCGAGGGAAAATCCGTTCG |
| 34 | AGAP002593 | ENST28106_F | TAATACGACTCACTATAGGGTGTTTGTGGTGCTGGTGGCG | ENST28106_R | TAATACGACTCACTATAGGGACGGATGGATCGTTCGCTGC |
| 35 | AGAP011503 | ENST14959_F | TAATACGACTCACTATAGGGCCACCACCGGCAACTTCC | ENST14959_R | TAATACGACTCACTATAGGGCGACAGGTCTAGCTTGTGC |
| 36 | AGAP009146 | ENST21506_F | TAATACGACTCACTATAGGGCAACGATTGCCCTGCTATGG | ENST21506_R | TAATACGACTCACTATAGGGTGCACGAGCTAGCCTTTTCCG |
| 37 | AGAP002186 | ENST06309_F | TAATACGACTCACTATAGGGCAAGAATTGAGCGTGCTGGTATGG | ENST06309_R | TAATACGACTCACTATAGGGCACATCAAGCTGTCGCTCATCA |
| 38 | ENSANGESTG00000008518 | ENST25832_F | TAATACGACTCACTATAGGGAATCCAGGAACCAAGCACCCG | ENST25832_R | TAATACGACTCACTATAGGGCGGGCACCTTCAGTTGCTCTTC |
| 39 | AGAP007314 | ENST16687_F | TAATACGACTCACTATAGGGCCGACGGGCCGTTATGTTGA | ENST16687_R | TAATACGACTCACTATAGGGTTCGCTTGCTGAACGGCTCAG |
| 40 | AGAP006275 | ENST21680_F | TAATACGACTCACTATAGGGCTGCAGGAGCTTTGCCGTAA | ENST21680_R | TAATACGACTCACTATAGGGAGCTTGCTTTCCACGCACTGC |
| 41 | AGAP008923 | ENST21979_F | TAATACGACTCACTATAGGGAAGAACTCAACACGTACGGC | ENST21979_R | TAATACGACTCACTATAGGGCTGCATCACGAACATTCCG |
| 42 | AGAP009184 | ENST22610_F | TAATACGACTCACTATAGGGAAGGCTCGTCTCGTTTCCATCG | ENST22610_R | TAATACGACTCACTATAGGGCGGCGCAACTAGAGTTTGACGA |
| 43 | AGAP009311 | ENST28550_F | TAATACGACTCACTATAGGGCACCGCATCGACGACGATTA | ENST28550_R | TAATACGACTCACTATAGGGTCAAACACCCAGCCCTGGTTG |
| 44 | AGAP003304 | ENST14526_F | TAATACGACTCACTATAGGGATACTCAAACAACAGGGAGCA | ENST14526_R | TAATACGACTCACTATAGGGCCTTATGCACCAGGTAG |
| 45 | AGAP002267 | ENST21854_F | TAATACGACTCACTATAGGGCACCACCACCACCATCATCGAC | ENST21854_R | TAATACGACTCACTATAGGGAAGCTTTCCCCACACTCGTCCG |
| 46 | AGAP011387 | ENST11371_F | TAATACGACTCACTATAGGGTGGTGGCGGTGGGCTT | ENST11371_R | TAATACGACTCACTATAGGGTGCAACCAGCTGACCCAG |
| 47 | AGAP000615 | ENST19196_F | TAATACGACTCACTATAGGGCCGGTTCGAGGAATCGTTCATG | ENST19196_R | TAATACGACTCACTATAGGGACGGCGCTCTTTCAGTGATTCC |
| 48 | AGAP009817 | ENST15861_F | TAATACGACTCACTATAGGGAATGGATGTTCCCGGCCTCC | ENST15861_R | TAATACGACTCACTATAGGGCGTTTGTCGGAAGGCCAGAACT |
| 49 | AGAP009642 | ENST22240_F | TAATACGACTCACTATAGGGTCGGTGACGATGTGTTCT | ENST22240_R | TAATACGACTCACTATAGGGCCGCTCCACCAGGAAGG |
| 50 | AGAP000911 | ENST21427_F | TAATACGACTCACTATAGGGATGAACGTGTACGAGACGAT | ENST21427_R | TAATACGACTCACTATAGGGACGGCTGCGATCGTT |
| 51 | AGAP005693 | ENST21822_F | TAATACGACTCACTATAGGGCTGCAAACGCTTGACCTTT | ENST21822_R | TAATACGACTCACTATAGGGAGCGTGATCAGCTTGTCA |
| 52 | AGAP007313 | ENST22987_F | TAATACGACTCACTATAGGGAAGGCAGCTTCTTCGGACTGGC | ENST22987_R | TAATACGACTCACTATAGGGACAAATCCCTCGTCACGCCG |
| 53 | AGAP001798 | ENST18359_F | TAATACGACTCACTATAGGGTACTGTTCCGAAATTCCCATCA | ENST18359_R | TAATACGACTCACTATAGGGCTCGATGGCGGCAATGTC |
| 54 | AGAP001242 | ENST13862_F | TAATACGACTCACTATAGGGCCAGCTGCATTGCCGTGTA | ENST13862_R | TAATACGACTCACTATAGGGATCACACGGCCGCCCTTGTA |
| 55 | AGAP009859 | ENST14861_F | TAATACGACTCACTATAGGGTACCGATCGTTCCCGTGGCA | ENST14861_R | TAATACGACTCACTATAGGGTTGTATCGCTGCGTCGTCGG |
| 56 | AGAP004557 | ENST12199_F | TAATACGACTCACTATAGGGTTGAGTCATTGTGGCAATTTTG | ENST12199_R | TAATACGACTCACTATAGGGTCGGAAGTGATCGATCAGATA |
| 57 | AGAP001148 | ENST12087_F | TAATACGACTCACTATAGGGAGCGGAGCAAGTGCAGA | ENST12087_R | TAATACGACTCACTATAGGGTATTGCTGCTAGTTGGAG |
| 58 | AGAP006377 | ENST13072_F | TAATACGACTCACTATAGGGCTGGACGCTGCTCTATCGCTCA | ENST13072_R | TAATACGACTCACTATAGGGCTCCTTCGCTATGGCACGTTC |
| 59 | AGAP003220 | ENST14719_F | TAATACGACTCACTATAGGGAGAAGTTTTTCGACTGCGA | ENST14719_R | TAATACGACTCACTATAGGGCGGGCGCAGGATCCAG |
| 60 | AGAP003473 | ENST14362_F | TAATACGACTCACTATAGGGAAATACGTGTTAGTCGCCCTGATGC | ENST14362_R | TAATACGACTCACTATAGGGCCTTCATCAGGAGACATGCTGTCG |
| 61 | AGAP010325 | ENST24749_F | TAATACGACTCACTATAGGGCTTATGGTGCAGCACAATG | ENST24749_R | TAATACGACTCACTATAGGGTGCCGCAACAGCTAAGG |
| 62 | AGAP007665 | ENST21542_F | TAATACGACTCACTATAGGGCGGAGTTGCAACAGAC | ENST21542_R | TAATACGACTCACTATAGGGTTGAACACGGTGTCCAGAT |
| 63 | AGAP003304 | ENST17918_F | TAATACGACTCACTATAGGGATACTCAAACAACAGGGAGCA | ENST17918_R | TAATACGACTCACTATAGGGCCTTATGCACCAGGTAG |
| 64 | AGAP008813 | ENST01123_F | TAATACGACTCACTATAGGGAGCCATACCTACAAATGAATGCCG | ENST01123_R | TAATACGACTCACTATAGGGTTTAGGACTGACCGGTTCCGTTTC |
| 65 | AGAP012432 | ENST00667_F | TAATACGACTCACTATAGGGTGACTCTGTTCCATGTTCTATT | ENST00667_R | TAATACGACTCACTATAGGGCCATTTGAATTTCAAACCAAACAA |
| 66 | AGAP010934 | ENST27303_F | TAATACGACTCACTATAGGGTTCACGGTGGCGATACGCAA | ENST27303_R | TAATACGACTCACTATAGGGCGCACGATTCCATGCAAACG |
| 67 | AGAP005761 | ENST04406_F | TAATACGACTCACTATAGGGCTTACTTCGGACCGGGT | ENST04406_R | TAATACGACTCACTATAGGGTACAGTACGATCGCATTCAT |
| 68 | AGAP008500 | ENST19960_F | TAATACGACTCACTATAGGGTCGAAAATAGTGGGCCAAGA | ENST19960_R | TAATACGACTCACTATAGGGCACGATCGACGCGACAA |
| 69 | AGAP003534 | ENST23319_F | TAATACGACTCACTATAGGGACATCACAAGCACCGCTAGCCA | ENST23319_R | TAATACGACTCACTATAGGGCCATCTCACGCACACTGCATCA |
| 70 | AGAP005888 | ENST14131_F | TAATACGACTCACTATAGGGAAACTGACCAGTCCGCCCG | ENST14131_R | TAATACGACTCACTATAGGGTCCCCTAGTGTCCGTGCCCA |
| 71 | AGAP009259 | ENST12287_F | TAATACGACTCACTATAGGGACCAGCGCCGAGTTCTAC | ENST12287_R | TAATACGACTCACTATAGGGCAGCTTCACGTACAGATCCT |
| Rel2 | AGAP006747 | Rel2_T7_F | TAATACGACTCACTATAGGGCCGCTACCAGTCGGAGATGCACGG | Rel2_T7_R | TAATACGACTCACTATAGGGCTCCACCCGGTACGCCCGGAAG |
| Cac | AGAP007938 | CACT_T7_F | TAATACGACTCACTATAGGGAGAGTCCGCTCTACACATCAGCA | CACT_T7_R | TAATACGACTCACTATAGGGAGACCGTTCGGGTTAATGATGAC |
| Bint2 | AGAP010233 | BINT2_T7_F | TAATACGACTCACTATAGGGCATCCAATGATGCTGTTTGC | BINT2_T7_R | TAATACGACTCACTATAGGGAACCCAGCAGCAATCAAGTT |
| 8001a | AGAP008001 | AGAP008001a_T7_F | TAATACGACTCACTATAGGGATTACAACATCCAGAAGGAGTC | AGAP008001a_T7_R | TAATACGACTCACTATAGGGTAGTACTTGAGGACAGCAAG |
| 8001b | AGAP008001 | AGAP008001b_T7_F | TAATACGACTCACTATAGGGAGGGTACGGTGATTGAGGTG | AGAP008001b_T7_R | TAATACGACTCACTATAGGGCTGTGCCTTCTCCTTTACGC |
| IAP1 | AGAP007294 | IAP1_T7_F | TAATACGACTCACTATAGGGACCGGAGTACTTCCACATCG | IAP1_T7_R | TAATACGACTCACTATAGGGTGGCAGTTGCTGTACCAGA |
| 5160 | AGAP005160 | AGAP005160_T7_F | TAATACGACTCACTATAGGGCTTCTCTGTCGATTCACCC | AGAP005160_T7_R | TAATACGACTCACTATAGGGTCTACTTGCTGCTTCCCGTT |
| LacZ |  | LacZ_T7_F | TAATACGACTCACTATAGGGAGAATCCGACGGGTTGTTACT | LacZ_T7_R | TAATACGACTCACTATAGGGCACCACGCTCATCGATAATTT |
|  | AGAP010592 | AgS7_qF | GTGCGCGAGTTGGAGAAGA | AgS7_qR | ATCGGTTTGGGCAGAATGC |
|  | AGAP004016 | 2_QF | CCAAGCACGGGAGTACATTT | 2_QR | TGTGCAACGCCTTGTATAGG |
|  | AGAP004928 | A14_QF | GGAAGCATCGAAAAGAGTGC | A14_QR | CCGCAAAATTTACCCCTTTT |
|  | AGAP005227 | A18_QF | CCACAAGCACGAGATTGAAA | A18_QR | ACATTGCCCACACTGTCAAA |
|  | AGAP009201 | A32_QF | AGGTCGGACTCGAAGGCTA | A32_QR | TGGACAGGTTGGGCAGAT |
