# Supplementary material for: Comprehensive Genetic Dissection of the Hemocyte Immune Response in the Malaria Mosquito Anopheles gambiae
Source: PLoS Pathog. 2013 Jan 31;9(1):e1003145. doi: 10.1371/journal.ppat.1003145 (PMC3561300; doi:10.1371/journal.ppat.1003145)
Supplement: Table S2 — Genes exploited as controls in viability assays. Name of dsRNA (#), AGAP code (IDs), gene name, IPRO ID and length of T7 dsRNA products are reported. (DOC) [file ppat.1003145.s008.doc]

**Table S2.** DsRNA exploited as controls in viability assays.

| *#* | *IDs* | *gene* | *IPRO description* | *IPRO* | *T7 (bp)* |
| --- | --- | --- | --- | --- | --- |
| IAP1 | AGAP007294 | IAP1 | Prot_inh_I32_IAP | IPR001370 | 559 |
| 8001a | AGAP008001 | - | Ubiquitin.like | - | 147 |
| 8001b | AGAP008001 | - | Ribosomal_S8E | IPR001047 | 344 |
| 5160 | AGAP005160 | - | GTPase_Rho | IPR003578 | 489 |
