# Supplementary material for: Comprehensive Genetic Dissection of the Hemocyte Immune Response in the Malaria Mosquito Anopheles gambiae
Source: PLoS Pathog. 2013 Jan 31;9(1):e1003145. doi: 10.1371/journal.ppat.1003145 (PMC3561300; doi:10.1371/journal.ppat.1003145)
Supplement: Table S3 — Phagocytosis assay results. Datasets from in vitro measurements using microplate reader were statistically analysed employing two statistical approaches, z-score threshold and ANOVA calculation. In column “#” are listed dsRNA labels; AGAP ID number and IPRO domain descriptions are reported in the next two columns. “Z-score” column lists genes with significant values at the indicated TP (for at least two replicates out of three) from plate reader measurements. Microplate reader values were also averaged and compared to dsLacZ control values: positive hits for each TP, according to ANOVA statistical analysis followed by Tukey's Multiple Comparison Test, are listed in “ANOVA P<0,05” column. 8 genes selected from these 13 significant candidates were evaluated in in vivo assay. The “in vivo % TP2” column reports the percent of phagocytosis as calculated by imaging analysis in in vivo experiments 2 h after challenge. SP, Signal Peptide; TD, transmembrane domain; ns, not significant; nd, not determined. (DOC) [file ppat.1003145.s009.doc]

**Table S3.** Phagocytosis assay. Results from *in vitro* screen (microplate reader) and *in vivo* assay.

### KDs decreasing the uptake of bioparticles

| *#* | *ID* | *IPRO domains/homologies* | *z-score* | *ANOVA P<0.05* | *in vivo % (TP2)* |
| --- | --- | --- | --- | --- | --- |
| A1 | AGAP000182 | SP, peptidase domains | ns | TP1-0, TP3-0, TP6-0, TP24-0 | 45.91+/-11.30 |
| A7 | AGAP002243 | Ankyrin repeat | ns | TP1-0 | 29.57+/-7.958 |
| A10 | AGAP003879 | TD, v-ATPase | ns | TP1-0 | 85.69+/-13.63 |
| A14 | AGAP004928 | TD, zinc finger, LPS-induced TNFa (eiger) | ns | TP1-0 | 58.88+/-17.06 |
| A37 | AGAP011223 | Fibrinogen (FBN8, FREP57, Dong Y., 2009) | TP3-1, TP24-6, TP24-0, TP24 | TP24-0 | 74.62+/-4.396 |
| #25 | AGAP000095 | TD, DUF590 | TP3-1, TP3-0, TP6-0 | TP3-0, TP6-0 | nd |
| #68 | AGAP008500 | TD | TP6-0 | ns | nd |

### KDs increasing the uptake of bioparticles

| *#* | *AGAP ID* | *IPRO domains/homologies* | *z-score* | *ANOVA P<0.05* | *in vivo % (TP2)* |
| --- | --- | --- | --- | --- | --- |
| A22 | AGAP006769 | SP, GO:0005515 | TP1-0, TP24-0 | TP6-0, TP24-0 | 112.1+/-21.95 |
| A26 | AGAP008492 |  | ns | TP6-0 | 153.2+/-30.97 |
| A33 | AGAP009459 | Protein kinase | ns | TP6-0 | 86.29+/-37.27 |
| #28 | AGAP001381 | EGF-like, laminin domains | TP3, TP6 | ns | nd |
| #10 | AGAP006914 | Fibrinogen (FBN30, FREP8, Dong Y., 2009) | ns | TP24-0 | nd |
| #11 | AGAP011197 | Fibrinogen/Lipocalin Q6VFG8 (FBN9, FREP13) | TP24, TP24-6, TP24-0 | TP24-0 | nd |
