# Supplementary material for: Comprehensive Genetic Dissection of the Hemocyte Immune Response in the Malaria Mosquito Anopheles gambiae
Source: PLoS Pathog. 2013 Jan 31;9(1):e1003145. doi: 10.1371/journal.ppat.1003145 (PMC3561300; doi:10.1371/journal.ppat.1003145)
Supplement: Table S4 — Luciferase assay results. Gene knockdowns (KD) that modulate the regulation of CEC and LRIM1 promoters upon PGN challenge according to the z-score analysis are shown; KDs modulating basal LRIM1 promoter activity (PBS challenge) are also summarized; IPRO domains short descriptions are reported. SP, Signal peptide; TD, transmembrane domain. (DOC) [file ppat.1003145.s010.doc]

**Table S4.** Luciferase assay summary.

**KDs modulating CEC1 and LRIM1 promoter activity (PGNvsPBS challenges)**

| *increasing CEC promoter activity* | | *decreasing CEC promoter activity* | |
| --- | --- | --- | --- |
| KD | IPRO domains | KD | IPRO domains |
| #8, AGAP006771 | TD, TNF-like | #13, AGAP009231 | TD, ninjurin |
| #31, AGAP010531 | SP, FBN12, FREP2 | A33*, AGAP009459 | kinase |

| *increasing LRIM1 promoter activity* | | *decreasing LRIM1 promoter activity* | |
| --- | --- | --- | --- |
| KD | IPRO domains | KD | IPRO domains |
| #6, AGAP009762 | EGF-like (Nimrod) | #28, AGAP001381 | EGF-like/laminin |
| #60, AGAP003473 | TD |  |  |
| A6, AGAP001964 | serin protease, CLIP |  |  |
| A23*, AGAP007499 | TD, chloride channel |  |  |

**KDs modulating basal LRIM1 promoter activity (PBS challenge)**

| *KDs increasing LRIM1 promoter activity* | | *KDs decreasing LRIM1 promoter activity* | |
| --- | --- | --- | --- |
| KD | IPRO domains | KD | IPRO domains |
| #28, AGAP001381 | EGF-like/laminin | REL2 |  |
| #37, AGAP002186 | LDL receptor |  |  |
| A30, AGAP009119 | TPR repeats |  |  |

* indicates that the averaged value calculated from the three KD replicates was also significant (P<0.05) according to Dunnet’s multiple-comparison post-test following one-way ANOVA.
