# Supplementary material for: Comprehensive Genetic Dissection of the Hemocyte Immune Response in the Malaria Mosquito Anopheles gambiae
Source: PLoS Pathog. 2013 Jan 31;9(1):e1003145. doi: 10.1371/journal.ppat.1003145 (PMC3561300; doi:10.1371/journal.ppat.1003145)
Supplement: Table S5 — Summary of RNAi screens results. In column “#” are listed dsRNA labels; AGAP ID number and IPRO domain descriptions and homologies are reported in the next 2 columns. KD phenotypes of genes that gave a positive phenotype in at least one of the 4 assays are summarized in the next 4 columns. (DOC) [file ppat.1003145.s011.doc]

### Table S5. Summary of RNAi screens results.

|  |  |  | *Knockdown phenotype* | | | |
| --- | --- | --- | --- | --- | --- | --- |
| *#* | *Gene ID* | *IPRO domains/homologies* | *Phagocytosis* | *CEC1 expression upon PGN challenge* | *LRIM1 expression upon PGN challenge* | *LRIM1 basal expression* |
| #25 | AGAP000095 | TD, DUF590 | decrease |  |  |  |
| A1 | AGAP000182 | SP, peptidase domains | decrease |  |  |  |
| #28 | AGAP001381 | EGF-like, laminin domains | increase |  | decrease | increase |
| A6 | AGAP001964 | serin protease (CLIP) |  |  | increase |  |
| #37 | AGAP002186 | LDL receptor |  |  |  | increase |
| A7 | AGAP002243 | ankyrin repeat | decrease |  |  |  |
| #60 | AGAP003473 | TD |  |  | increase |  |
| A10 | AGAP003879 | TD, v-ATPase | decrease |  |  |  |
| A14 | AGAP004928 | TD (eiger) | decrease |  |  |  |
| A22 | AGAP006769 | SP, GO:0005515 | increase |  |  |  |
| #8 | AGAP006771 | TD, TNF-like |  | increase |  |  |
| #10 | AGAP006914 | Fibrinogen (FBN30, FREP8) | increase |  |  |  |
| A23 | AGAP007499 | TD, chloride channel |  |  | increase |  |
| A26 | AGAP008492 |  | increase |  |  |  |
| #68 | AGAP008500 | TD | decrease |  |  |  |
| A30 | AGAP009119 | TPR repeats |  |  |  | increase |
| #13 | AGAP009231 | TD, ninjurin |  | decrease |  |  |
| A33 | AGAP009459 | protein kinase | increase | decrease |  |  |
| #6 | AGAP009762 | EGF-like (Nimrod) |  |  | increase |  |
| #31 | AGAP010531 | SP, Fibrinogen (FBN12,FREP2) |  | increase |  |  |
| #11 | AGAP011197 | Fibrinogen (FBN9, FREP13) | increase |  |  |  |
| A37 | AGAP011223 | Fibrinogen (FBN8, FREP57) | decrease |  |  |  |
