# Supplementary material for: Comprehensive Genetic Dissection of the Hemocyte Immune Response in the Malaria Mosquito Anopheles gambiae
Source: PLoS Pathog. 2013 Jan 31;9(1):e1003145. doi: 10.1371/journal.ppat.1003145 (PMC3561300; doi:10.1371/journal.ppat.1003145)
Supplement: Text S1 — Additional information and data on viability assay, ex vivo phagocytosis assay, knockdown efficiency assessment and Drosophila melanogaster orthologs. (DOC) [file ppat.1003145.s012.doc]

**Supporting Information**

## **Viability assay**

Three genes were selected as controls for the optimization of viability assays in *An. gambiae* cell lines (Table S2). AGAP005160 is the homolog of the *Drosophila melanogaster* Rho1 small GTPase (CG8416, 94% identity and 98% similarity of protein sequence). Activation of Rho1 in *D. melanogaster* coordinates F-actin and microtubule remodelling, thereby enabling cytokinesis [1]. RNAi-mediated KD of *rho1* gene disrupts cytokinesis in S2 *Drosophila* cells [2,3]. Significant reduction in cell viability is not reported, but impairment of cell division and cytokinesis defects caused by KD produce an easy visual phenotype [4].

AGAP007294 is the putative *An. gambiae* homolog of D-IAP1 (*Drosophila* Inhibitor of Apoptosis 1, CG12284). This gene is essential to prevent inappropriate caspase activation and ubiquitous apoptosis. Moreover, it is precisely regulated through its ubiquination that is stimulated by Reaper-family proteins [5,6]. DIAP1 silencing by RNAi in cultured S2 *Drosophila* cells induces a time-dependent decrease in cell viability [4]. Whereas the molecular characterization of DIAP1 homologs has been already carried out in *Aedes* and *Culex* spp. mosquitoes [7,8], no experimental data are available so far to elucidate the role of predicted IAPs in *An. gambiae*. According to VectorBase (<http://agambiae.vectorbase.org/>), AGAP007294 is the *An. gambiae* homolog of DIAP1 (40% protein sequence identity, 57% similarity).

As an additional control, we selected the gene *AGAP008001*, the homolog of *D. melanogaster* RpS27A [9,10], a protein containing ubiquitin/ribosomal fusion. In addition to polyubiquitin peptides, ubiquitin-fusion proteins represent precursors of ubiquitin. Two ubiquitin/ribosomal protein fusions are described, namely UbL40 and UbS27, where the ubiquitin tag is fused with ribosomal proteins L40 and S27, respectively [11]. Ubiquitin-like dsRNA treatment causes a dramatic increase in cell mortality in *D. melanogaster*. The ubiquitin-like CG11700 may act in the same pathway as DIAP1 to directly prevent Nc-caspase activated apoptotic cell death. Moreover, KD of ribosomal protein S8 (CG7808) causes strong increase in *D. melanogaster* cell mortality [4]. Nearly all genes encoding ribosomal components exhibited severe lethal phenotypes when silenced in cell-based RNAi screens [4].

We initially targeted the control genes by RNAi in 8 different *An. gambiae* cell cultures. A fluorescence microscopy-based viability assay was performed. Briefly, following dsRNA challenges, cells in 96-well plates were allowed to grow for 4 days, and then dead cells were revealed following staining with Sytox Green dye. This nucleic acid stain easily penetrates cells with compromised plasma membranes but it is not able to cross membranes of living cells. Simultaneous staining with the cell permeable Hoechst nucleic acid dye allowed calculation of the total cell numbers at the moment of the assay. The assay showed that the toxicity caused by *LacZ* dsRNA treatment of the different cell lines was extremely variable, enabling the selection of the most suitable cell cultures for further screens, i.e. Sua5.1*, 4a3B, 4a2 and L3-5 (Figure S1). Moreover, we observed that the highest level of cell mortality was caused by treatment with ds*IAP1* in 6 cell lines out of the 8. Figure S1 shows representative images of L3-5 cells stained after dsRNA treatments.

To evaluate variations in cell growth and development, a protocol was also developed in ImageJ to compute size, shape and number of cell nuclei stained with Hoechst. Silencing of *AGAP005160* (*An. gambiae* homolog of Rho1 small GTPase) promotes both reduction of the number of living cells and significant increase in the nucleus size (Figure S2), suggesting that, after duplication of DNA, cells may not be able to complete their lifecycle due to cytokinesis impairment. This was an expected phenotype, as RNAi-mediated KD of *rho1* disrupts cytokinesis in *D. melanogaster* S2 cells [4].

## **Ex vivo phagocytosis assay**

For optimization of the phagocytosis assay, we used dsRNA against *Cactus* and *BINT2* (beta-integrin 2) [12], treatment of cells with Cytochalasin D (a permeable and potent inhibitor of actin polymerization) and incubation on ice as controls. The effect of control dsRNAs on phagocytosis, as revealed by microtiter readout, is reported in Figure S4A. We reproducibly observed an increase in Gram negative *Escherichia coli* phagocytosis efficiency (as well as an opposite inhibitory effect of Gram positive *Staphylococcus aureus* uptake) after treatment with ds*Cactus*, even if to a smaller extent than reported previously [12]. As *Cactus* mutant *Drosophila* have an overabundance of hemocytes [13], a similar effects of *Cactus* KD in *An. gambiae* could have masked the results of our phagocytosis assay. To address this possibility, we carried out a cell viability assay that showed no significant variations in cell viability and number. No significant inhibition of *E. coli* bioparticles uptake after treatment with ds*BINT2* was observed, in contrast to what has been previously shown [12]. Finally, significant inhibition of phagocytosis was detected following treatment of cells with Cytochalasin D (Figure S4B).

## **KD efficiency assessment**

We assessed the KD efficiency of 4 randomly selected genes 4 days after treatment of Sua 5.1* cells with respective dsRNA using quantitative RT-PCR (Figure S5). KD efficiency was highly reproducible between the replicates but varied between different genes from ~90% to ~50% (Figure S5).

## **Drosophila melanogaster orthologs and their phenotypes in cell-based RNAi screens**

The presence of *D. melanogaster* orthologs of the 109 *An. gambiae* genes examined here was assessed. According to VectorBase, 60 genes have fly orthologs (see Dataset S1). We compared the RNAi phenotypes of these genes with the RNAi phenotypes of their *D. melanogaster* orthologs as presented in the GenomeRNAi database [14,15]. 14 of the 60 genes showed phenotype(s) in one or more of our RNAi screens, and of these 11 also exhibited phenotype(s) in fly RNAi screens. Interestingly, the phenotypes of these 11 genes were comparable between the two insects. Of the remaining 46 orthologs, 33 exhibited phenotype(s) in at least one of the *Drosophila* RNAi screens.

**References**

1. Gonzalez C (2003) Dispatch. Cell division: the place and time of cytokinesis. Curr Biol 13: R363-365.

2. Echard A, Hickson GR, Foley E, O'Farrell PH (2004) Terminal cytokinesis events uncovered after an RNAi screen. Curr Biol 14: 1685-1693.

3. Somma MP, Fasulo B, Cenci G, Cundari E, Gatti M (2002) Molecular dissection of cytokinesis by RNA interference in Drosophila cultured cells. Mol Biol Cell 13: 2448-2460.

4. Boutros M, Kiger AA, Armknecht S, Kerr K, Hild M, et al. (2004) Genome-wide RNAi analysis of growth and viability in Drosophila cells. Science 303: 832-835.

5. Hay BA, Wassarman DA, Rubin GM (1995) Drosophila homologs of baculovirus inhibitor of apoptosis proteins function to block cell death. Cell 83: 1253-1262.

6. Steller H (2008) Regulation of apoptosis in Drosophila. Cell Death Differ 15: 1132-1138.

7. Beck ET, Blair CD, Black WCt, Beaty BJ, Blitvich BJ (2007) Alternative splicing generates multiple transcripts of the inhibitor of apoptosis protein 1 in Aedes and Culex spp. mosquitoes. Insect Biochem Mol Biol 37: 1222-1233.

8. Blitvich BJ, Blair CD, Kempf BJ, Hughes MT, Black WC, et al. (2002) Developmental- and tissue-specific expression of an inhibitor of apoptosis protein 1 homologue from Aedes triseriatus mosquitoes. Insect Mol Biol 11: 431-442.

9. Barrio R, del Arco A, Cabrera HL, Arribas C (1994) Structure and expression of the Drosophila ubiquitin-80-amino-acid fusion-protein gene. Biochem J 302 ( Pt 1): 237-244.

10. Lee HS, Simon JA, Lis JT (1988) Structure and expression of ubiquitin genes of Drosophila melanogaster. Mol Cell Biol 8: 4727-4735.

11. Catic A, Ploegh HL (2005) Ubiquitin--conserved protein or selfish gene? Trends Biochem Sci 30: 600-604.

12. Moita LF, Wang-Sattler R, Michel K, Zimmermann T, Blandin S, et al. (2005) In vivo identification of novel regulators and conserved pathways of phagocytosis in A. gambiae. Immunity 23: 65-73.

13. Qiu P, Pan PC, Govind S (1998) A role for the Drosophila Toll/Cactus pathway in larval hematopoiesis. Development 125: 1909-1920.

14. Horn T, Arziman Z, Berger J, Boutros M (2007) GenomeRNAi: a database for cell-based RNAi phenotypes. Nucleic Acids Res 35: D492-497.

15. Gilsdorf M, Horn T, Arziman Z, Pelz O, Kiner E, et al. (2010) GenomeRNAi: a database for cell-based RNAi phenotypes. 2009 update. Nucleic Acids Res 38: D448-452.
